# The role of high cell density in the promotion of neuroendocrine transdifferentiation of prostate cancer cells

**DOI:** 10.1186/1476-4598-13-113

**Published:** 2014-05-20

**Authors:** Zuzana Pernicová, Eva Slabáková, Radek Fedr, Šárka Šimečková, Josef Jaroš, Tereza Suchánková, Jan Bouchal, Gvantsa Kharaishvili, Milan Král, Alois Kozubík, Karel Souček

**Affiliations:** 1 Department of Cytokinetics, Institute of Biophysics, Academy of Sciences of the Czech Republic, v.v.i, Královopolská 135, CZ-612 65 Brno, Czech Republic; 2 Center of Biomolecular and Cellular Engineering, International Clinical Research Center, St. Anne’s University Hospital Brno, Brno, Czech Republic; 3 Department of Experimental Biology, Faculty of Sciences, Masaryk University, Brno, Czech Republic; 4 Department of Histology and Embryology, Faculty of Medicine, Masaryk University, Brno, Czech Republic; 5 Laboratory of Molecular Pathology and Institute of Molecular and Translational Medicine, Faculty of Medicine and Dentistry, Palacky University Olomouc, Olomouc, Czech Republic; 6 Department of Urology, Faculty of Medicine and Dentistry, Palacky University Olomouc, Olomouc, Czech Republic

**Keywords:** Neuroendocrine transdifferentiation, Androgen depletion, High cell density, Cell cycle arrest, cAMP signaling

## Abstract

**Background:**

Tumor heterogeneity and the plasticity of cancer cells present challenges for effective clinical diagnosis and therapy. Such challenges are epitomized by neuroendocrine transdifferentiation (NED) and the emergence of neuroendocrine-like cancer cells in prostate tumors. This phenomenon frequently arises from androgen-depleted prostate adenocarcinoma and is associated with the development of castration-resistant prostate cancer and poor prognosis.

**Results:**

In this study, we showed that NED was evoked in both androgen receptor (AR)-positive and AR-negative prostate epithelial cell lines by growing the cells to a high density. Androgen depletion and high-density cultivation were both associated with cell cycle arrest and deregulated expression of several cell cycle regulators, such as p27^Kip1^, members of the cyclin D protein family, and Cdk2. Dual inhibition of Cdk1 and Cdk2 using pharmacological inhibitor or RNAi led to modulation of the cell cycle and promotion of NED. We further demonstrated that the cyclic adenosine 3′, 5′-monophosphate (cAMP)-mediated pathway is activated in the high-density conditions. Importantly, inhibition of cAMP signaling using a specific inhibitor of adenylate cyclase, MDL-12330A, abolished the promotion of NED by high cell density.

**Conclusions:**

Taken together, our results imply a new relationship between cell cycle attenuation and promotion of NED and suggest high cell density as a trigger for cAMP signaling that can mediate reversible NED in prostate cancer cells.

## Background

Prostate cancer is one of the leading causes of cancer in men worldwide. Although the growth of both normal prostate epithelial cells and cancer cells is dependent on the presence of androgens, chemical or surgical androgen depletion therapy is the mainstay of treatment for metastatic prostate disease. However, in many patients an androgen-independent (castration-resistant) form of prostate cancer develops within 18–24 months. Castration-resistant prostate cancer (CRPC) is currently an incurable stage with poor prognosis [1]. During attempts to find new treatment modalities for CRPC it has been hypothesized that one of the events contributing to the development of anti-androgen resistance is neuroendocrine transdifferentiation (NED) of prostate cancer cells (summarized in [2]). NED thus serves as an example of one of the many levels of tumor heterogeneity and cancer cell plasticity that in general represent a challenging issue for effective clinical diagnosis and therapy [3].

In prostate carcinoma there is an increase in the number of cells with neuroendocrine-like properties over time. Because normal NE cells are thought to be post-mitotic [4], it is proposed that new cells with NE-like properties originate through the process of NED from pre-existing epithelial cancer cells [5]. Such cancer cells acquire a NE-like phenotype—they are able to secrete several neuropeptides and are androgen-independent. It was shown that NED can be induced *in vitro* by various stimuli, such as androgen depletion [6,7], increased levels of interleukin-6 (IL-6) [8], activation of Wnt [9] and EGF [10] signaling pathways, activation of the cyclic adenosine 3′, 5′-monophosphate (cAMP) signaling pathway [11-13], or ionizing radiation [14,15]. In addition, several genes and transcription factors were shown to be involved in NED, for example protocadherin-PC and the transcription factors Foxa2 and NeuroD1 (summarized in [2]).

Androgen depletion, which induces NED, is associated with cell cycle arrest in G1 phase [16,17]. This cell cycle arrest is linked to modulation of well-known cell cycle regulators involved in G1 phase progression and the G1 to S phase transition [16,18]. Another mechanism that contributes to cell cycle arrest is the phenomenon of contact inhibition. High-density cultivation is associated with arrest in G1 phase that is accompanied by decreased Cdk2 and Cdk4 activity, even in cancer cells that are refractory to the typical contact inhibition exhibited by normal cells. Furthermore, cell density can also influence intracellular signaling, as shown by density-dependent changes in intra- and extra-cellular distribution of cAMP [19].

In the present study, we focused on the role of cell cycle modulation in the regulation of NED in prostate cancer cells. We showed that androgen depletion and cell cycle modulation mediated by high cell density both promoted NED, which was demonstrated by increased expression of characteristic markers both in AR-positive and AR-negative prostate epithelial cell lines of different origin. We identified an important role of Cdk1 and Cdk2 activity in promoting NED by cell cycle attenuation. Finally, our results suggest a role of cAMP signaling activation in NED promotion by high cell density in AR-positive prostate cancer cell lines. Taken together, our data identify a novel condition leading to the promotion of NED in prostate cancer cells and define specific molecular mechanisms that determine this process.

## Results

### Androgen depletion and high cell density promote NED characteristics of prostate cancer cells

NED markers have diverse biologic functions: γ-enolase is one of the iso-enzymes of the glycolytic enzyme enolase, which catalyzes the conversion of 2-phospho-glycerate to phosphoenolpyruvate and is found in mature neurons (summarized in [20]); cytoskeletal protein tubulin β-III is an early marker of neuronal differentiation [21]; chromogranin A is a prohormone expressed in endocrine cells and peptidergic neurons that mediates granule formation (summarized in [22]); and L-dopa decarboxylase is an enzyme involved in the synthesis of dopamine, serotonin, and tryptamine that was shown to interact with androgen receptor (AR) [23]. We decided to assess several different markers of NED at both the protein and mRNA level because the expression of different NED markers may not correlate in every experimental set-up, as was shown for γ-enolase and chromogranin A in LNCaP cells undergoing NED [24].

**Figure 1:**
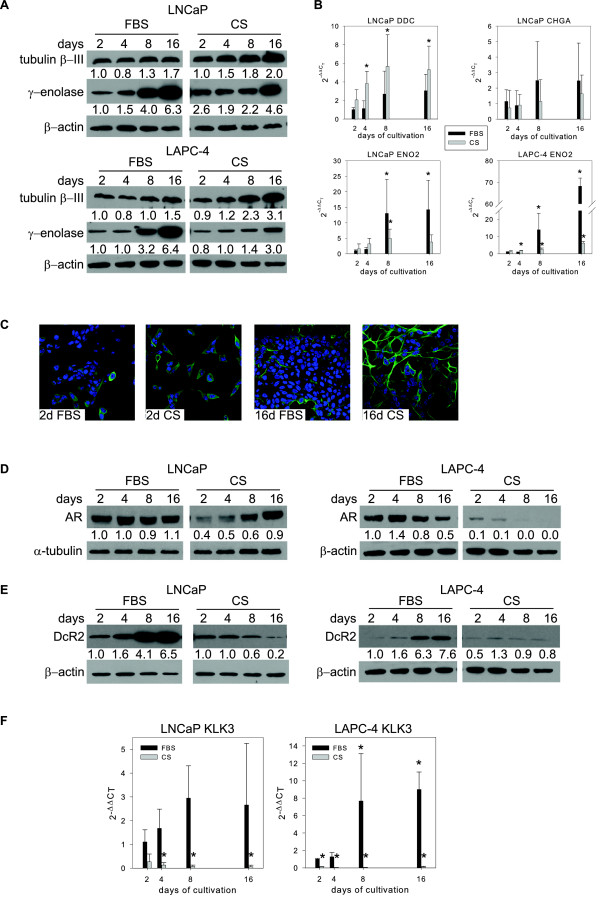
**Androgen depletion and high cell density both promote neuroendocrine transdifferentiation (NED) of LNCaP and LAPC-4 cells independent of AR activity.** LNCaP and LAPC-4 cells were cultivated for 2 to 16 days in the presence (FBS) or absence (CS) of androgens in the cultivation medium. **A**, Western blot analysis of the expression of γ-enolase and tubulin β-III. **B**, qRT-PCR analysis of changes in mRNA levels of L-dopa decarboxylase (DDC), chromogranin A (CHGA), and γ-enolase (ENO2). The bars represent means ± standard deviation (SD) from three independent experiments. **C**, Immunofluorescence detection of tubulin β-III expression in LNCaP cells. **D**, Western blot analysis of changes in expression of androgen receptor (AR) in LNCaP and LAPC-4 cells. AR activity was assessed by **(E)** western blot analysis of protein levels of androgens-regulated protein tumor necrosis factor receptor superfamily member 10D (DcR2), and **(F)** qRT-PCR analysis of changes in mRNA levels of the prostate-specific antigen (KLK3). The data represent means ± SD of three independent experiments. “*”denotes statistical significance (P < 0.05) compared with control (2 days in FBS).

Androgen depletion in LNCaP cells increased protein levels of the widely used NED markers γ-enolase, tubulin β-III [25] (Figure 1A, right panel) and mRNA levels of the NED markers γ-enolase (ENO2) and aromatic L-amino-acid decarboxylase (DDC) [26] (Figure 1B). Surprisingly, NED markers were also up-regulated at both protein and mRNA levels in cells cultivated at high density in the presence of androgens (FBS, day 8 and 16). Importantly, similar effects of androgen depletion and high density in promoting NED were observed in another prostate cancer cell line, LAPC-4 (Figure 1A, B). Immunofluorescence analysis of tubulin β-III expression showed a positive signal in LNCaP cells cultivated for 16 days under androgen-depleted conditions in dextran/charcoal-stripped serum-containing medium (16d CS) or under high cell density conditions after cultivation for 16 days in FBS (16d FBS), where the signals were detected mainly on the edges of high-density areas (Figure 1C).

Contradictory results have been published regarding the reversibility of NED in prostate cancer cells [6,27]. Therefore, we investigated whether NED promoted by androgen depletion or high cell density is a reversible or irreversible process. The expression of γ-enolase decreased in both cell lines when cells cultivated for 16 days in the presence of FBS were re-seeded into FBS at low density and cultivated for another 4 to 8 days (Additional file 1: Figure S1A). However, splitting CS-cultivated cells (after 16 days) from CS to either FBS or CS at low density did not decrease the androgen depletion-enhanced protein level of γ-enolase during further cultivation for up to 8 days after re-seeding. This may imply that the NED phenotype is reversible when evoked by high cell density (FBS), but irreversible when promoted by androgen depletion (CS).

### High cell density promotes NED in both AR-positive and AR-negative prostate epithelial cell lines

The regulation of NED by high cell density in both LNCaP (androgen-sensitive) and LAPC-4 (androgen-dependent) cells led us to propose the hypothesis that cultivation at a high cell density may modulate the expression and activity of AR. In LNCaP cells, high cell density did not affect the protein level of AR (Figure 1D, left panel). Androgen depletion initially induced a decrease in the protein level of AR but after 16 days of cultivation AR expression returned to a level comparable to that of the control. In contrast, AR expression was decreased in LAPC-4 cells in response to high cell density or androgen depletion (Figure 1D, right panel). Next, we analyzed changes in expression levels of downstream targets of AR, namely the tumor necrosis factor receptor superfamily member 10D (decoy receptor 2, DcR2), expression of which is regulated by androgens [28,29], and prostate-specific antigen (PSA, encoded by the KLK3 gene). In both cell lines tested, high cell density cultivation (FBS) was associated with an increase in DcR2 protein expression and up-regulation of KLK3 mRNA level (Figures 1E and F, respectively) implying increasing activity of AR in this model. As expected, expression of both androgen-regulated genes was inhibited when cells were cultivated in the absence of androgens (CS), which is indicative of decreasing AR activity. Taken together, these results showed that high cell density-induced NED promotion in AR-positive prostate cancer cell lines LNCaP and LAPC-4 in comparison to CS-induced NED is not associated with the inhibition of AR activity.

To further strengthen our results obtained from AR-expressing prostate cancer cell lines, we performed high-density cultivation experiments with the AR-negative prostate cancer cell lines PC3 and DU-145 (Additional file 1: Figure S1B-D). Cultivation of PC3 and DU-145 at high density for 10 days led to up-regulation of the NED marker γ-enolase at both the protein and mRNA level (Additional file 1: Figure S1B, D). Moreover, we observed a similar increase in γ-enolase protein in PC3 cells stably transfected with AR [30] (Additional file 1: Figure S1C) when cultivated at high density (Additional file 1: Figure S1B). Concurrently, the mRNA for AR-regulated gene for DcR2 (TNFRSF10D) was increased in response to high cell density cultivation (Additional file 1: Figure S1E). These results support our hypothesis that the promotion of NED in response to high density may occur in both AR-positive and AR-negative prostate epithelial cell lines.

**Figure 2:**
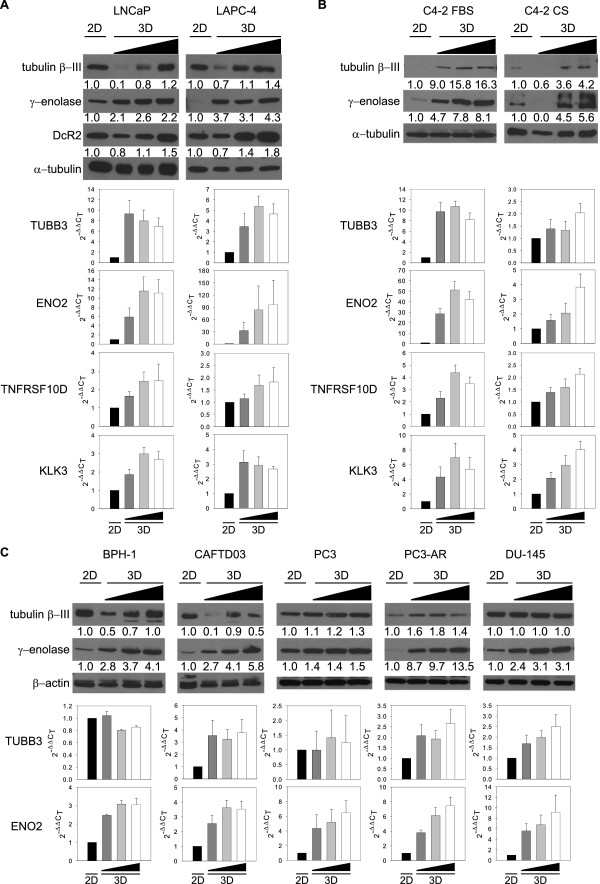
**High cell density promotes NED of prostate cell lines cultured in 3D conditions independent of AR status. A**, Analysis of NED marker expression in LNCaP and LAPC-4 cells seeded on Alvetex® scaffold at increasing density (0.5 × 10^6^, 1.0 × 10^6^, and 1.5 × 10^6^). Western blot (upper) and qRT-PCR (lower) analysis of the NED markers γ-enolase (ENO2) and tubulin β-III (TUBB3). Activity of AR was examined by detection of expression of the androgen-regulated protein DcR2 at protein and mRNA level (TNFRSF10D) and PSA at mRNA level (KLK3) in 3D conditions. **B**, Western blot (upper) and qRT-PCR (lower) analysis of NED markers in the C4-2 cell line (LNCaP androgen-independent clone) in 3D conditions. Activity of AR was confirmed by detection of DcR2 at both protein and mRNA level (TNFRSF10D) and PSA at mRNA level (KLK3). **C**, Changes in protein (upper) and mRNA (lower) levels of NED markers after 3D cultivation of AR-negative prostate cell lines BPH-1, CAFTD03, PC3, DU-145, and PC3 cells stably transfected with AR (PC3-AR). qRT-PCR data are presented as mean ± S.D. of two independent experiments except for results for C4-2, which are from one experiment. Triangle represents increasing seeding density in 3D conditions on Alvetex (0.5 × 10^6^, 1.0 × 10^6^, and 1.5 × 10^6^, respectively).

The effect of high density on NED promotion was also assessed during growth under three-dimensional (3D) conditions using an Alvetex® scaffold (Additional file 2: Figure S2A). Western blot analysis showed that expression of NED markers tubulin β-III and γ-enolase in LNCaP and LAPC-4 cells increased at both the protein and mRNA level with increasing seeding density in 3D conditions (Figure 2A). Confocal microscopy confirmed that cells cultivated in a 3D scaffold expressed the NED marker tubulin β-III (Additional file 3: Figure S2B). Changes in AR activity, assessed by expression of its downstream targets DcR2 (TNFRSF10D gene) and PSA (KLK3 gene) at the protein and/or mRNA level (Figure 2A), were similar to those of high density-promoted NED in 2D conditions (Figure 1E, F). Moreover, similar results were obtained for C4-2 cells, an androgen-independent subline of LNCaP, in both the presence (FBS) and absence (CS) of androgens (Figure 2B).

Again, we observed that high density in 3D conditions evoked NED in both AR-positive and AR-negative cell lines, as in 2D conditions (Figure 1 and Additional file 1: Figure S1B-D), because cultivation in 3D at high density also increased the expression of NED markers at both protein and mRNA level in the AR-negative prostate cell line BPH-1 and its tumorigenic clone CAFTD03, as well as in the prostate cancer cell lines PC3 and DU-145 (Figure 2C). Similar to results obtained for 2D culture, γ-enolase was up-regulated in PC3 cells stably expressing AR in 3D conditions. Moreover, when comparing different seeding densities on Alvetex scaffold, mRNA level for DcR2 gene TNFRSF10D increased in 3D conditions in PC3-AR cell line (Additional file 1: Figure S2C).

To confirm the uniformity of AR activity in response to high cell density at a single cell level, we used the Stellaris® RNA FISH technique to assess KLK3 mRNA expression *in situ*. As shown in Additional file 3: Figure S3A, the higher frequency of KLK3 transcript per cell was detected in high-density cultivation (12 days FBS) in compare with low density cultivation (2 days FBS) especially in LNCaP cells. Heterogeneity of AR activity in LAPC-4 cells in low high cell density condition is comparable. On the other hand, the signal was totally absent after cultivation in androgen-depleted conditions (12 days CS). These trends correlate with the KLK3 qRT-PCR analysis (Figure 1F). Next, we detected prostate specific membrane antigen (PSMA) using flow cytometry (Additional file 3: Figure S3B). Consistent with published data showing that PSMA is repressed by androgens [31], we detected decreased expression of PSMA in both LNCaP and LAPC-4 cells after 8 days of cultivation at high density in complete medium (8 days FBS) compared with androgen-depleted cultivation (8 days CS). We did not detect any subpopulations or significant differences in the heterogeneity of PSMA expression using flow cytometry. We can conclude that the activity of AR in high-density conditions is higher or comparable with low density condition and significantly higher in compare with androgen-depleted cultivation.

In summary, these results show that high density and androgen depletion both increase the expression of NED markers in prostate cancer cell lines. Interestingly our data demonstrated capability of high cell density to promote NED in both AR-positive and AR-negative cell lines in both 2D and 3D condition. Moreover NED promotion in the high cell density condition was paralleled with increased activity of AR in AR-positive cell lines.

### Promotion of NED in response to high cell density or androgen depletion is accompanied by cell cycle arrest

**Figure 3:**
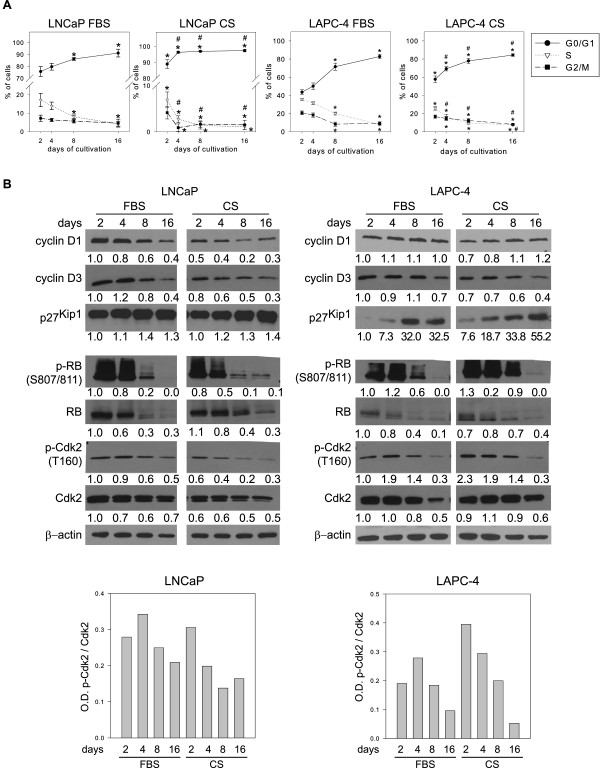
**Androgen depletion and high cell density both induce cell cycle arrest in LNCaP and LAPC-4 cells. A**, Analysis of changes in cell cycle distribution in response to high cell density (FBS) and androgen depletion (CS). The data represent means ± SD of three independent experiments. “*”denotes statistical significance compared with control (2 days in FBS), “^#^” denotes statistical significance compared with 2 days in CS. **B**, Western blot analysis of the expression of selected cell cycle regulators in LNCaP and LAPC-4 cells. Graphs represent optical density (O.D.) of p-Cdk2 normalized to O.D. of total Cdk2.

Long-term androgen depletion led to arrest of LNCaP and LAPC-4 cells in the G0/G1 phase of the cell cycle (Figure 3A). Although induction of cell cycle arrest by high cell density was slower than that induced by androgen depletion, cells were significantly arrested in the G0/G1 phase in both the androgen-depleted and high-density models. Based on this observation, we further examined expression levels of cyclin D family proteins, which are important factors in the transition through G1 phase. As shown in Figure 3B, cyclin D1 was down-regulated in response to high density and androgen depletion in LNCaP cells but not in LAPC-4 cells. In contrast, cyclin D3 was down-regulated in both cell lines in both models of NED promotion. We were unable to detect cyclin D2 in LNCaP or LAPC-4 cells (data not shown). We also analyzed expression of the cyclin-dependent kinase inhibitors p27^Kip1^ and p21^Cip1^. As shown in Figure 3B, p27^Kip1^ levels increased in response to high cell density or androgen depletion whereas p21^Cip1^ levels decreased in both models of NED promotion (data not shown). In both models, the cell cycle arrest observed in the G0/G1 phase correlated with decreased expression of phosphorylated retinoblastoma protein (Rb) and decreased total level of Rb protein (Figure 3B). The Rb protein can be phosphorylated on Ser 807/811 by different cyclin dependent kinases (Cdk) including Cdk4 and Cdk2 [32,33]. As Cdk2 is an important regulator of G1/S transition, we next examined the activity and expression of Cdk2 in our experimental models. We detected phosphorylation of Cdk2 at Thr160, which is crucial for the activation of Cdk2, and showed that the levels of total and phosphorylated Cdk2 decreased in response to androgen depletion and high cell density (Figure 3B).

Taken together, these results showed that androgen depletion and high cell density modulate cell cycle machinery and induce cell cycle arrest in a similar fashion.

To check if the correlation between decreased proliferation and promotion of NED is also observed *in vivo* in human cancer tissue, we examined the expression of NED markers γ-enolase and chromogranin A and the proliferation marker Ki-67 by immunohistochemical examination of formalin-fixed paraffin-embedded tissue samples from 18 patients with advanced prostate adenocarcinoma (patients information Additional file 4: Table S3; subset of patients with lymph node metastases was selected in order to obtain a higher percentage of NED for evaluation). There was a slight trend towards an inverse correlation between Ki-67 score and γ-enolase expression (p = 0.146), suggesting that tumors with slower proliferation (Ki-67 low) have higher expression of the NED marker γ-enolase (Additional file 5: Figure S4).

### Deregulation of cell cycle by inhibition of Cdk1 and/or Cdk2 activity leads to NED promotion

Next, we focused on elucidating the mechanism underlying the promotion of NED by high cell density. Given that both androgen depletion and high cell density evokes cell cycle arrest, we hypothesized that modulation of the cell cycle machinery is an important event in promoting NED in prostate cancer cells.

First, we addressed the role of cyclin D1 and D3 in the promotion of NED. Using RNA interference approach, we down-regulated the expression of cyclin D1, cyclin D3, or both, in LNCaP and LAPC-4 cells. A decrease in cyclin D1 and/or D3 protein levels led to significant modulation of the cell cycle; however, modulation of cell cycle progression by cyclin D1 and D3 down-regulation was not sufficient for NED promotion in AR-positive LNCaP and LAPC-4 cells (Additional file 6: Figure S5).

**Figure 4:**
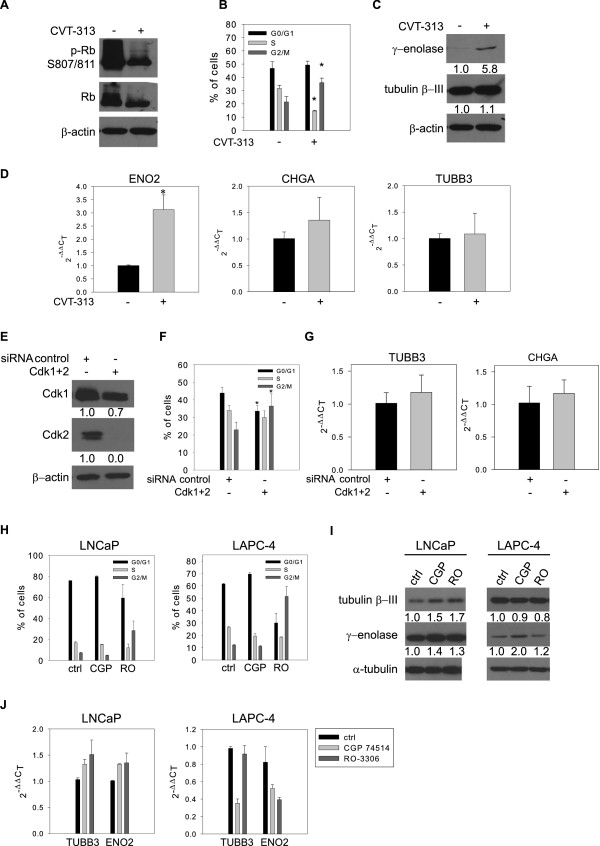
**Deregulation of the cell cycle by inhibition of Cdk1 and/or Cdk2 boosts expression of NED markers in AR-positive prostate cancer cell lines.** A-D, LAPC-4 cells were treated with an inhibitor of Cdk2 activity, CVT-313 (10 μM, 48 h). **A**, Western blot analysis of Rb protein. **B**, Analysis of cell cycle distribution. The data represent means ± SD (n=4). **C**, Western blot analysis of expression of γ-enolase and tubulin β-III. **D**, qRT-PCR analysis of TUBB3, CHGA, and ENO2 mRNA level. The data represent means ± SD (n=4). “*” denotes statistical significance compared with control. **E**-**G**, LAPC-4 cells were transfected with control siRNA or with a combination of Cdk1- and Cdk2 siRNAs for 48 hours. **E**, Western blot analysis of Cdk1 and Cdk2 48 hours after transfection. **F**, Analysis of cell cycle distribution in response to Cdk1 and Cdk2 down-regulation. The data represent means ± SD (n=3). **G**, qRT-PCR analysis of changes in mRNA levels of TUBB3 and CHGA in response to Cdk1 and Cdk2 down-regulation. The data represent means ± SD (n=4). “*”denotes statistical significance compared with control siRNA. **H**-**J**, LNCaP and LAPC-4 cells were treated with DMSO or the indicated Cdk1 inhibitors for 48 hours (LNCaP: 2 μM CGP, 7.5 μM RO; LAPC-4: 3.5 μM CGP, 7.5 μM RO). **H**, Analysis of cell cycle distribution in LNCaP and LAPC-4 cells treated with the indicated Cdk1 inhibitors. The data are presented as means ± SD of one out of two independent experiments performed in duplicate. **I**, Western blot analysis of γ-enolase and tubulin β-III in response to Cdk1 inhibitors. **J**, qRT-PCR analysis of TUBB3 and ENO2 in response to Cdk1 inhibitors. The data are presented as means ± SD of one out of two experiments performed in duplicate. CGP, CGP 74514A; RO, RO-3306.

Because androgen depletion and high cell density both decreased the expression and activity of Cdk2 and increased expression of the Cdk2 inhibitor p27^Kip1^, we next focused on the role of these molecules in deregulation of the cell cycle and the subsequent promotion of NED. Using a p27^Kip1^ RNAi approach we were unable to detect any changes in the cell cycle in LAPC-4 cells, although p27^Kip1^ was effectively down-regulated in these cells (data not shown). We therefore focused on Cdk2 and used a reversible ATP-competitive inhibitor, CVT-313, to inhibit Cdk2 activity. Efficient inhibition of Cdk2 activity by CVT-313 was demonstrated by de-phosphorylation of Ser 807/811 in the Rb protein, a downstream target of Cdk2 (Figure 4A). This was accompanied by deregulation of the cell cycle, as reflected by a decrease in the percentage of cells in S-phase and a significant arrest of cells in G2/M phase (Figure 4B). Importantly, this down-regulation of Cdk2 activity and cell arrest in the G2/M phase was followed by up-regulation of the NED marker γ-enolase at both protein and mRNA levels (Figures 4C, D).

Next, we performed experiments using specific siRNA to down-regulate expression of Cdk2. RNAi led to significant down-regulation of Cdk2 protein level but had no significant effects on the cell cycle in LNCaP and LAPC-4 cells (data not shown). Because CVT-313 inhibitor was shown to also inhibit Cdk1 activity in complex with cyclin B (IC50 4.2 μM) [34], we next performed experiments using specific siRNAs against Cdk1 or Cdk2 to elucidate whether the effects observed with CVT-313 treatment were caused by decreased activity of both Cdk1 and Cdk2. Co-transfection of LAPC-4 cells with Cdk1- and Cdk2-specific siRNAs led to decreased levels of both proteins to a different degree (Figure 4E). This was associated with modulation of the cell cycle, as documented by the increased percentage of cells in G2/M phase after transfection with the siRNAs (Figure 4F). Next, we investigated whether this cell cycle deregulation affected NED promotion. Detection of mRNA levels of selected NED markers showed a trend of increased expression of TUBB3 (118%) and CHGA (117%) (Figure 4G). To further investigate the role of Cdk1 inhibition in promoting NED we inhibited Cdk1 using two selective inhibitors, CGP 74514A and RO-3306 (Figure 4 H-J). Treatment with these inhibitors in non-toxic concentrations led to deregulation of the cell cycle in both LNCaP and LAPC-4 cell lines, particularly after treatment with RO-3306 (Figure 4H). This cell cycle deregulation was associated with a trend of increased expression of the NED markers γ-enolase and tubulin β-III at the protein and/or mRNA level (Figure 4I,J) in LNCaP cells, but not in LAPC-4.

In summary, these results showed that deregulation of the cell cycle by inhibition of the activity of the upstream signaling molecules Cdk1 and Cdk2 is at least partly involved in NED modulation in AR-positive prostate cancer cell lines LNCaP and LAPC-4.

### cAMP signaling is involved in NED promotion in response to high cell density

**Figure 5:**
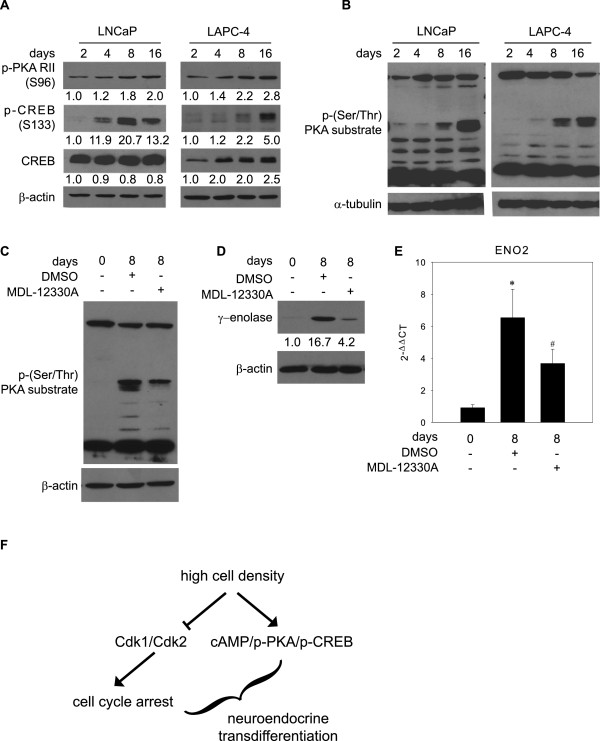
**Involvement of the cAMP signaling pathway in NED promoted by high cell density.** Western blot analysis of changes in expression of selected members of the cAMP signaling pathway in response to high cell density. **A**, Phosphorylation of PKA RII (Ser 96), phosphorylation of CREB (Ser 133), and total level of CREB were detected in cells cultivated in complete media with androgens (FBS) for 2 to 16 days. **B**, Analysis of p-(Ser/Thr) PKA substrates in cells cultivated in the presence of androgens for 2 to 16 days. **C**, Analysis of the p-Ser/Thr PKA substrate in LAPC-4 cells in response to treatment with the MDL-12330A, an inhibitor of cAMP signaling. Cells were treated with 2 μM MDL-12330A for 8 days. **D**, Analysis of the expression of the NED marker γ-enolase in response to treatment with MDL-12330A. **E**, qRT-PCR analysis of mRNA levels of ENO2 in response to inhibition of cAMP signaling. The data represent means ± SD of three experiments. “*” and “^#^” denote statistical significance compared with control cells harvested on day 0 or cells cultivated for 8 days and treated with vehicle, respectively. **F**, Schematic illustration of the proposed mechanism for the promotion of NED by high cell density.

Finally, we aimed to identify the signaling pathway responsible for NED promotion in response to high cell density. It has previously been shown that high cell density is associated with changes in the extracellular to intracellular distribution of cAMP, which acts as a second messenger in G-protein coupled pathways [19]. Moreover, it is known that cAMP signaling is involved in the promotion of NED [11,35]. Therefore, we investigated the involvement of cAMP signaling in NED promoted by high cell density. To assess the status of this pathway, we examined changes in phosphorylation (activation) of the cAMP-dependent protein kinase A regulatory subunit 2 (PKA RII) and cAMP responsive element-binding (CREB) protein in response to high cell density. As shown in Figure 5A, levels of both p-CREB (Ser133) and p-PKA RII (Ser96) increased in response to high cell density in LNCaP and LAPC-4 cells. To confirm that PKA, the key downstream molecule in cAMP signaling, is activated by high cell density, we measured the global pattern of phosphorylation of PKA substrates by western blot analysis. The activity of PKA was increased in both LNCaP and LAPC-4 cells after prolonged periods of cultivation at high cell density, as documented by an increase in overall phosphorylation of PKA substrates (Figure 5B). Next, we focused on functional validation of the involvement of cAMP signaling in NED promoted by high cell density using an irreversible inhibitor of adenylate cyclase, MDL-12330A. Cells were cultivated for 8 days in FBS with MDL-12300A or vehicle. Long-term treatment of LAPC-4 cells with MDL-12330A led to inhibition of cAMP/PKA signaling, as confirmed by inhibition of the global phosphorylation pattern of PKA substrates (Figure 5C). This inhibition of cAMP signaling led to a significant inhibition of NED, as confirmed by decreased protein and mRNA levels of the NED marker, γ-enolase (Figures 5D and E). These results confirmed our hypothesis that cAMP signaling is involved in NED evoked by high cell density in AR-positive prostate cancer cells.

## Discussion

To our knowledge, this is the first demonstration that the plasticity of prostate cancer cells enables promotion of NED by modulation of the cell cycle in conditions of high cell density. It has been shown that NED can be induced predominantly by androgen depletion [6,7], but also by a wide variety of other stimuli, including IL-6 [8], Wnt(s) [9], EGF [10], or cAMP signaling [11,12,27]. Androgen deprivation therapy (ADT) represents a standard treatment for advanced prostate cancer [36]. The action of androgens is predominantly mediated through AR and its co-activators, which have been shown to be critical regulators of the G1 to S transition in prostate cancer cells [16,17]. The important role of cyclin D1 and cyclin D3 in this process was demonstrated by Xu et al. [37]. However, the link between ADT-induced NED and mechanisms of cell cycle regulation remains unclear. Here, we showed that androgen depletion and high cell density both led to the promotion of NED and modulation of the cell cycle machinery in a very similar manner, although inhibition of AR activity was confirmed only in androgen-depleted conditions and not in the high cell density condition. We demonstrated that Cdk1 and/or Cdk2 inhibition, but not cyclin D1 or D3 down-regulation, is sufficient for NED promotion in the presence of androgens. More importantly, we identified a key role of the cAMP/PKA signaling pathway in NED promoted by high cell density. Our study defines a novel mechanism highlighting how high tumor density could modulate the plasticity of prostate cancer cells and influence disease progression.

In our previous study, we demonstrated that both androgen depletion and high cell density cultivation led to increased expression of cytokeratins, general markers of epithelial differentiation [38]. Because androgen deprivation is a well-known promoter of NED [39], we compared the expression of NED markers between prostate cancer cells cultured at high density and cells cultured in the absence of androgens. Surprisingly, androgen depletion and high cell density both promoted increase in expression of several NED markers (γ-enolase, tubulin β-III, and aromatic L-amino acid decarboxylase) in prostate cancer cells. The effect of high density on NED promotion is androgen-independent, since similar regulation of NED markers was observed in C4-2 cells, an androgen-independent sub-line of LNCaP. Moreover, a similar high density-induced increase in the level of NED markers was detected using the AR-negative prostate cell line BPH-1 and its tumorigenic derivative CAFTD03, as well as the cancer cell lines DU-145 and PC3. Furthermore, re-introduction of AR into AR-negative PC3 cells did not have any effect on high cell density-induced NED. This phenotype was also confirmed in a 3D culture system in all above mentioned cell lines. Moreover, we observed a slight trend towards the correlation between decreased proliferation and NED promotion in a small cohort of patients with advanced prostate cancer. This is in contrast to previously published data showing that a high Ki-67 labeling index is weakly associated with high chromogranin A expression (Spearman’s correlation 0.164) [40]. However, the authors of that study showed no correlation between Ki-67 labeling index and expression of AR or NeuroD1, another neuroendocrine marker. NeuroD1 was previously shown to be expressed in aggressive prostate cancer cell lines and prostate cancer samples, although co-expression with chromogranin A was found only rarely [41]. Bubendorf et al. [42] did not found significant association between Ki-67 and neuroendocrine differentiation while Grobholz et al. [43] observed higher proliferation index (assessed by Ki-67) in tumors with large clusters of NE differentiation in comparison to negative tumors or with solitary NE cells. With respect to our observations (both in-vitro and in our small patient cohort) and varying results in the literature, we may state that the neuroendocrine transdifferentiation may be present in tumors with slower proliferation in some patients.

Because the correlation of NED with cell cycle arrest had been described in several experimental models [35,44], we next focused on modulation of the cell cycle machinery. We showed that the induction of cell cycle arrest by androgen depletion is associated with down-regulation of cyclin D1 protein in LNCaP cells but not in LAPC-4 cells, and with down-regulation of cyclin D3 in both cell lines. Similar effects were observed when NED was promoted by high cell density. These results are in accordance with previously published data showing that androgen-induced proliferation of prostate cancer cells is accompanied by increased levels of D-type cyclins [37] and, conversely, androgen deprivation causes down-regulation of protein levels of cyclin D1 and cyclin D3 in LNCaP cells [16,37]. RNAi-mediated down-regulation of cyclin D1 in LNCaP cells and cyclin D3 in LAPC-4 cells led to modulation of the cell cycle and an increased percentage of cells in the G0/G1 phase; however, this was not associated with the promotion of NED. Thus, we conclude that although the down-regulation of D-type cyclins leads to cell cycle arrest in the G0/G1 phase, it is not sufficient for promoting NED in AR-positive prostate cancer cells.

Next, we focused on the role of the more general cell cycle regulator Cdk2. Both the activity and expression of Cdk2 were down-regulated during NED promotion by androgen depletion and high cell density, and this down-regulation correlated with up-regulation of the Cdk inhibitor p27^Kip1^ in both of our models. It was previously shown that Cdk2 expression and activity together with Rb protein phosphorylation, are regulated by androgens [16,45]. Generally, inhibition of Cdk activity causes cell cycle arrest and inhibits proliferation of prostate cancer cells. Our results showed that inhibition of Cdk2 causes cell cycle attenuation, in particular accumulation in the G2/M phase in LAPC-4 cells; this arrest is associated with significant promotion of NED. These results, which indicate a functional role of the inhibition of Cdk2 activity in the regulation of NED, are supported by the findings of other investigators. For example, it has been reported that silibinin-induced NED in LNCaP cells is also associated with cell cycle arrest and decreased Cdk2 levels [46]. We did not observe a significant change in the expression of NED markers when Cdk2 expression was reduced using a siRNA-mediated approach (data not shown). However, transfection of Cdk2 siRNA in combination with Cdk1 siRNA resulted in a slight trend towards NED promotion. This observation is in agreement with results obtained using CVT-313 inhibitor, which at the applied dose preferentially inhibits Cdk2, but might also partially inhibit Cdk1 [34]. To reveal the role of Cdk1 in NED promotion we used selective inhibitors. Treatment with subtoxic concentrations of the Cdk1-specific inhibitor CGP 74514 only slightly modulated the cell cycle, whereas RO-3306 caused accumulation of cells in G2/M phase, in accordance with previously published data [47]. This was accompanied by increased expression of NED markers at the protein and mRNA level in LNCaP cells but not in LAPC-4 cells, which only showed up-regulation of γ-enolase expression at the protein level. Further studies of the mechanisms by which Cdk1 and Cdk2 are involved in the plasticity of prostate cancer cells are necessary based on the fact that different approaches to the modulation of their expression and activity were not uniformly reflected in terms of NED promotion. Since experiments for elucidating the involvement of Cdk1 and Cdk2 in promoting NED were performed only in AR-positive prostate cancer cell lines, investigating the AR-negative cell lines might shed more light in proposed role of Cdk1 and Cdk2 deregulation in promoting NED. Our observations suggest that the association between NED and the cell cycle, and the role of particular regulators of cell cycle machinery is more complex and also cell type-dependent. The clinical potential of pharmacological inhibition of Cdk activity in cancer therapy has been demonstrated in several studies (for review see [48]). However, based on our observations, it is important to consider the possible effects of Cdk1 and Cdk2 inhibition in NED promotion in prostate cancer cells.

It has previously been shown that NED can be induced by physiological and pharmacological agents that elevate intracellular cAMP levels [27]. Treatment of prostate cancer cells with cAMP leads to changes in the expression of Hox genes located at the HOXD locus [13] including the Neuro D1 transcription factor, which is expressed in malignant NE cells [41]. Moreover, other downstream targets of cAMP, PKA [11] and CREB [14], are directly involved in NED. Furthermore, promotion of NED by cAMP-inducing agents is a reversible process. Interestingly, it has been shown that the cAMP level can be modulated by cell density [19]. Based on these facts, we hypothesized that the promotion of NED by high cell density can be mediated by the activation of cAMP signaling. Our results demonstrated that cAMP signaling is indeed activated in response to high cell density, as demonstrated by increased levels of downstream target molecules of cAMP, such as phosphorylated PKA regulatory subunit II and phosphorylated CREB. Interestingly, cAMP inhibits proliferation of breast cancer cells via increased expression of p27^Kip1^ and decreased activity of Cdk2 [49]. These observations are indirectly in accordance with our observation, since in response to high cell density we observed increased activation of cAMP-mediated signaling, increased p27^Kip1^, and decreased Cdk2 expression. Moreover, it was shown that cAMP inhibits Cdk2 activity and Rb phosphorylation in adipose stem cells [50]. More importantly, the functional involvement of cAMP was confirmed by the demonstration that treatment of prostate cancer cells with MDL-12330A, a potent inhibitor of adenylate cyclase, abolished the promotion of NED by high cell density. In summary, these experiment support our hypothesis that activation of cAMP signaling mediates NED promotion by high cell density in AR-positive prostate cancer cell lines (Figure 5F). Based on our results we conclude that modulation of the cell cycle by high cell density can promote reversible NED in prostate epithelial cancer cells. Our study also suggests that prostate cancer tissue remodeling, in association with disease progression or therapy, might contribute to tumor progression by modulating the plasticity of cancer cells and by promoting NED.

## Conclusions

We have demonstrated a new relationship between high cell density, cell cycle attenuation, and promotion of NED and suggest high cell density as a trigger for cAMP signaling that can mediate reversible NED in prostate cancer cells.

## Materials and Methods

### Cell culture and treatment

LNCaP cells (DSMZ) [51] (androgen-sensitive cell line carrying mutation in the gene encoding AR [52]), and LAPC-4 cells (androgen-dependent cell line carrying WT gene encoding AR [53]) were cultivated as described previously [38]. Culture conditions for the androgen-independent subline C4-2 [54] were similar to those for the parental LNCaP cell line. Under experimental conditions (high-density NED promotion, siRNA transfection, treatment with inhibitors), LNCaP cells were cultivated with 5% FBS or 5% dextran/charcoal-stripped FBS (CS, for androgen depletion), and LAPC-4 cells were cultivated with 10% FBS and 1 nM R1881, or with 10% CS. BPH-1 cells [55] and the BPH-1 tumorigenic clone CAFTD03 [56] were cultivated as described previously [57]. PC3 (ATCC) and PC3 cells stably expressing AR [30] were cultivated in F12 with 10% FBS and penicillin and streptomycin. DU-145 cells (ATCC) were cultivated in RPMI 1640 with 10% FBS and penicillin and streptomycin. AmpFLSTR® Identifiler® PCR Amplification Kit (Life Technologies) was used to verify the origin of cell lines.

To evoke NED, LNCaP and LAPC-4 cells were cultivated as follows: cells were seeded at a density of 20,000/cm^2^ in the appropriate complete medium with FBS (day -1). After 24 hours, the medium was exchanged for medium with FBS or CS (day 0). Cells were continuously cultivated for 2 to 16 days without splitting, but with exchange of the medium for fresh medium twice a week. Cells were collected for further analysis on days 2, 4, 8, and 16 after the change of medium on day 0.

For cultivation in 3D conditions, we used Alvetex® polystyrene scaffold inserts in 6-well plates (AVP004), 12-well plates (AVP002), or 24-well plates (AVP006) containing 200 μm thick Alvetex polystyrene scaffold (Reinnervate). Cells were seeded at a density of 0.5 × 10^6^, 1.0 × 10^6^, and 1.5 × 10^6^ cells per insert and cultivated for 72 to 96 hours with regular media exchanges. Cells that were seeded on standard Petri dishes in standard media and at standard seeding densities and cultivated for either 1 day or 4 days were used as a 2D control. Experiments in 3D were performed with two independent repetitions.

For the inhibition of Cdk2 activity we used a selective ATP-competitive Cdk2 inhibitor III [CVT-313, 2 (*bis*-(Hydroxyethyl) amino)-6-(4-methoxybenzylamino)-9-isopropyl-purine)] (#238803 Merck). For inhibition of Cdk1 activity we used the Cdk1 inhibitor CGP 74514A [N-(cis-2-Aminocyclohexyl) -N-(3-chlorophenyl)-9-ethyl-9H-purine-2,6-diamine, #217696, Calbiochem] and ATP-competitive Cdk1 Inhibitor IV RO-3306 [(5Z)-2-((Thiophen-2-yl)methylamino)-5-((quinolin-6-yl) methylene) thiazol-4(5H)-one, #217699, Calbiochem], both dissolved in DMSO. For inhibition of cAMP signaling we used an adenylate cyclase-specific inhibitor MDL-12330A hydrochloride [*N*- (*cis-*2-phenyl-cyclopentyl) azacyclotridecan-2-imine-hydrochloride, M-182, Sigma-Aldrich]. For all treatments, LNCaP and LAPC-4 cells were seeded at a density of 20,000 or 30,000 cells/cm^2^ in appropriate media (IMDM + 10% FBS + 1 nM R1881 + antibiotics for LAPC-4, RPMI + 5% FBS + antibiotics for LNCaP cells). After 48 hours, cells were treated with the indicated concentrations of selected inhibitors and control cells were treated with the equivalent concentration of dMSO (not exceeding 0.1%). Cells were collected for further analysis 48 hours after treatment. All experiments were performed at least twice with technical duplicates.

### Cell cycle analysis

Cells were fixed, stained, and analyzed by flow cytometry using FACSCalibur™ or BD FACSVerse (Becton Dickinson) as described previously [33]. At least two independent repetitions were performed for each experiment.

### Cell transfection and RNA interference

LNCaP and LAPC-4 cells were transfected with small inhibitory RNA (siRNA) duplexes (Santa Cruz Biotechnology) directed against non-targeting control (sc-37007), Cdk1 siRNA (sc-29252), and Cdk2 siRNA (sc-156139) using the Neon® Transfection System (Life Technologies). Transfection was performed in a 10-μl tip according to the manufacturer’s recommendations. Cells were harvested 48 hours after transfection for further analysis. Experiments were performed in three independent repetitions.

### RNA isolation and real-time reverse transcription polymerase chain reaction (qRT-PCR)

Total RNA was isolated using High Pure RNA Isolation Kit (Roche). PCR was performed using the One Step SYBR® PrimeScript™ RT-PCR Kit II (Perfect Real Time) according to the manufacturer’s recommendations on a RotorGene 6000 (Corbett Research) [33]. The sequences of the primers used are listed in Additional file 7: Table S1-A. Changes in gene expression were calculated using the comparative threshold cycle method, with POLR2A as a normalizing gene [58]. Data from at least three experiments were normalized for each gene using the mean C_T_ value for the control sample (2 days of incubation with FBS, control siRNA, vehicle-treated cells, or cells harvested at day 0). Alternatively, two-step qRT-PCR was performed. Up to 1 μg of isolated RNA was reverse transcribed to cDNA with the High Capacity RNA-to-cDNA Kit (Applied Biosystems). qRT-PCR was performed on a Light Cycler 480 (Roche) using the Light Cycler 480 Master Mix in combination with Human Universal Probe Library (Roche). Primer and probe combinations used in assays are listed in Additional file 7: Table S1B. Results for genes of interest were normalized to the housekeeping gene POLR2a assessed using Light Cycler 480 software and are presented as $2^{-\Delta \Delta C_{T}}$. Experiments were performed in at least two independent repetitions or in technical duplicates.

### Electrophoresis and western blotting

Collected cell pellets were lysed and the protein extracts were separated and blotted as described previously [33]. The primary and secondary antibodies used are listed in Additional file 8: Table S2. Detection of α-tubulin and β-actin served as a control of equal loading. All western blots are presented as typical results of at least two independent repetitions. Densitometry analyses were performed using ImageJ software (NIH). Values given below a particular band represent normalized results of densitometry analysis of the given image (integrated density for the particular band was assessed and all values were normalized to the control of equal loading).

### Immunofluorescence microscopy

Cells were cultivated, fixed, permeabilized, and stained as described previously [38]. The primary and secondary antibodies used are listed in Additional file 8: Table S2. Fluorescence images of the cells were obtained using a confocal microscope (TSC SP5X, Leica Microsystems).

### Statistical analysis

Statistical analysis was performed using STATISTICA for Windows software (StatSoft). When the data variance was homogenous, one-way analysis of variance followed by the Fisher or Tukey range test was used. If the data variance was non-homogenous, the Mann-Whitney U-test was performed.

Supplementary Material and Methods can be found in Additional file 9.

## Abbreviations

AR: Androgen receptor; cAMP: Cyclic adenosine 3′, 5′-monophosphate; CS: Dextran/charcoal-stripped FBS; Cdk1: Cyclin-dependent kinase 1; Cdk2: Cyclin-dependent kinase 2; CREB: CAMP responsive element-binding; CRPC: Castration-resistant prostate cancer; DcR2: Tumor necrosis factor receptor superfamily member 10D (decoy receptor 2); NED: Neuroendocrine transdifferentiation; PKA: Protein kinase A; PSA: Prostate-specific antigen; PSMA: Prostate-specific membrane antigen; Rb: Retinoblastoma protein.

## Competing interests

The authors declare that they have no competing interests.

## Authors’ contributions

ZP carried out experiments, analyzed results and wrote the manuscript. JB and GK provided IHC and data analysis. MK handled selection of patient samples for IHC analysis. ES, RF, TS and ŠŠ carried out expression analysis and analyzed data. JJ performed automatic image analysis of mRNA FISH. AK revised the manuscript. KS carried out particular flow cytometry analyses, supervised the experimental work, participated in data analysis and interpretation of results, and wrote the manuscript. All authors read and approved the manuscript.

## Supplementary Material

Additional file 1: Figure S1 A, Induction of neuroendocrine transdifferentiation by high cell density, but not by androgen depletion, is a reversible process. Cells were cultivated for 16 days to induce NED as described in Material and Methods section. After 16 days the cells were re-seeded at a low density (10,000 cells/cm^2^) in the appropriate cultivation media. Cells grown in FBS were re-seeded into media with FBS; cells grown in CS were re-seeded into either CS or FBS. Cells were further cultivated for 2, 4, and 8 days (16+2, 16+4, and 16+8, respectively) without splitting and appropriate medium was exchanged with fresh one twice a week. Expression of the NED marker γ-enolase in response to re-seeding was assessed using western blot analysis. A typical result of three independent repetitions is presented. B-D, NED is promoted by high density also in AR-negative prostate epithelial cell lines. B, PC-3, PC3-AR, and DU-145 cells were cultivated as described in Supplementary Material and Methods. Expression of NED markers γ-enolase and tubulin β-III was assessed by western blot analysis. C, Western blot analysis of AR expression to confirm its presence in PC3-AR cells; LNCaP cells served as a positive control. Results from one repetition out of two performed in technical duplicate are presented. D, qRT-PCR analysis of the NED marker γ-enolase (ENO2) and tubulin β-III (TUBB3) in PC3, PC3-AR, and DU-145 cells cultivated as described. Results from two repetitions performed in technical duplicate are presented (n=4). E, qRT-PCR analysis of DcR2 gene (TNFRSF10D) in PC3-AR cells cultivated as described in Additional file 9. Results from two repetitions performed in technical duplicate are presented (n=4). Click here for file

Additional file 2: Figure S2 Cultivation of prostate cancer cell lines in 3D conditions using Alvetex scaffold. A, LNCaP and LAPC-4 cells were cultivated in 3D conditions using Alvetex® scaffold at the indicated seeding densities per insert in complete media. After 72 hours, live cells were visualized by staining with 0.5% neutral red solution (N6634, Sigma-Aldrich) according to the manufacturer’s protocol. Increased intensity of staining indicates increased cell density. B, Immunofluorescence detection of tubulin β-III expression in LNCaP and LAPC-4 cells after 3 days of cultivation on Alvetex® inserts. Staining was performed according to the manufacturer’s protocol. Specifications of the antibodies used are provided in Table S2. C, qRT-PCR analysis of DcR2 gene (TNFRSF10D) in PC3-AR cells cultivated in 3D conditions on Alvetex scaffold as described in Additional file 9. The triangle represents increasing seeding density in 3D conditions on Alvetex (0.5×10^6^, 1.0×10^6^, and 1.5×10^6^, respectively). Results from two independent repetitions are presented (n=2). Click here for file

Additional file 3: Figure S3 Assessment of AR activity at a single cell level after high-density cultivation and prolonged androgen ablation. A, Activity of AR in response to androgen depletion (12d CS) and at high density (12d FBS) assessed by detection of KLK3 mRNA using a mRNA FISH technique and quantified (B) as described in Additional file 9. n, number of identified nuclei C, Flow cytometric analysis of prostate membrane specific antigen (PSMA) in LNCaP and LAPC-4 cells in response to androgen depletion (8d CS) or high density (8d FBS). Staining was performed as described in Additional file 9. Representative results from one repetition out of two performed in replicate are presented. d, days. Click here for file

Additional file 4: Table S3 Characteristics of human prostate tumor samples. Subsets of patients with advanced CaP with lymph node metastases were selected to obtain a sufficient percentage of NED for statistical evaluation. dg, diagnosis; GS, Gleason score; pT, pathologic T stage; pN, positivity of lymph nodes; %, percentage of positive staining. Click here for file

Additional file 5: Figure S4 Immunohistochemical staining of formalin-fixed paraffin-embedded patient samples. A, Patients 2 and 9 display low Ki-67 expression (less than 30% nuclear positivity) and multiple chromogranin A- and γ-enolase-positive NE and/or NE-like cells. Patients 13 and 15 display high Ki-67 expression (more than 30% nuclear positivity) and single chromogranin A and γ-enolase-positive NE and/or NE-like cells (magnification 40×). B, Quantification of γ-enolase and chromogranin A expression in patient tumor samples. In total, 10 patients with low Ki-67 expression and 8 patients with high Ki-67 expression were examined. Information on the patients is provided in Table S3. Click here for file

Additional file 6: Figure S5 Modulation of the cell cycle by down-regulation of cyclin D1 and/or cyclin D3 does not lead to induction of NED in LNCaP and LAPC-4 cells. A, Western blot analysis of the efficiency of cyclin D1 and cyclin D3 down-regulation in LNCaP and LAPC-4 cells following transfection with control siRNA A or specific siRNA (20 or 40 nM). Experiments were performed as described in Supplementary Materials and Methods. B, Analysis of changes in cell cycle distribution in response to cyclin D1 and/or cyclin D3 down-regulation in LNCaP and LAPC-4 cells. Data represent means ± SD of three independent experiments. C, Western blot analysis of changes in protein levels of NED markers in response to cyclin D1 and/or cyclin D3 siRNA. It should be noted that other samples irrelevant to this study were analyzed on the same membranes. These samples were omitted from the pictures presented and the western blots presented here are therefore cropped. D, qRT-PCR analysis of changes in mRNA level of the NED marker γ-enolase (ENO2) in response to cyclin D1 and/or cyclin D3 siRNA. Data represent means ± SD of two independent experiments performed in duplicate (n=4). “*” and “^#^” denote statistical significance (P<0.05) compared with cells transfected with 20 nM or 40 nM control siRNA A, respectively. Click here for file

Additional file 7: Table S1 Sequences of primers used in quantitative RT-PCR. Click here for file

Additional file 8: Table S2 Specification of antibodies used for western blot analysis, immunofluorescence and flow cytometry. Click here for file

Additional file 9 Supplementary Material and Methods. Click here for file
