# Supplementary figures and images for: The role of high cell density in the promotion of neuroendocrine transdifferentiation of prostate cancer cells
Source: Mol Cancer. 2014 May 20;13:113. doi: 10.1186/1476-4598-13-113 (PMC4229954; doi:10.1186/1476-4598-13-113)

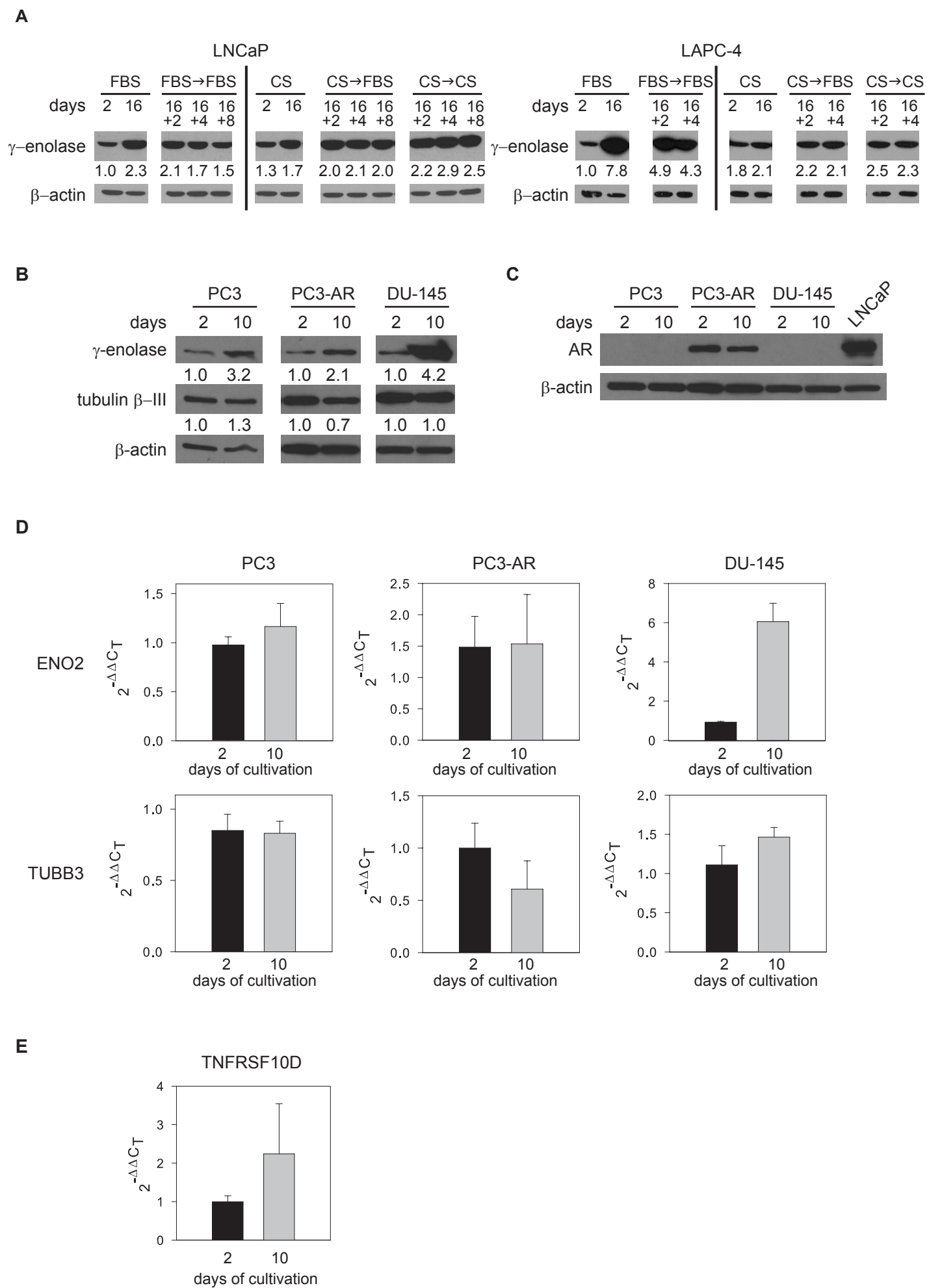

Supplementary Figure 1

Supplement: Additional file 1: Figure S1 — A, Induction of neuroendocrine transdifferentiation by high cell density, but not by androgen depletion, is a reversible process. Cells were cultivated for 16 days to induce NED as described in Material and Methods section. After 16 days the cells were re-seeded at a low density (10,000 cells/cm2) in the appropriate cultivation media. Cells grown in FBS were re-seeded into media with FBS; cells grown in CS were re-seeded into either CS or FBS. Cells were further cultivated for 2, 4, and 8 days (16+2, 16+4, and 16+8, respectively) without splitting and appropriate medium was exchanged with fresh one twice a week. Expression of the NED marker γ-enolase in response to re-seeding was assessed using western blot analysis. A typical result of three independent repetitions is presented. B-D, NED is promoted by high density also in AR-negative prostate epithelial cell lines. B, PC-3, PC3-AR, and DU-145 cells were cultivated as described in Supplementary Material and Methods. Expression of NED markers γ-enolase and tubulin β-III was assessed by western blot analysis. C, Western blot analysis of AR expression to confirm its presence in PC3-AR cells; LNCaP cells served as a positive control. Results from one repetition out of two performed in technical duplicate are presented. D, qRT-PCR analysis of the NED marker γ-enolase (ENO2) and tubulin β-III (TUBB3) in PC3, PC3-AR, and DU-145 cells cultivated as described. Results from two repetitions performed in technical duplicate are presented (n=4). E, qRT-PCR analysis of DcR2 gene (TNFRSF10D) in PC3-AR cells cultivated as described in Additional file 9. Results from two repetitions performed in technical duplicate are presented (n=4). [file 1476-4598-13-113-S1.pdf]

A

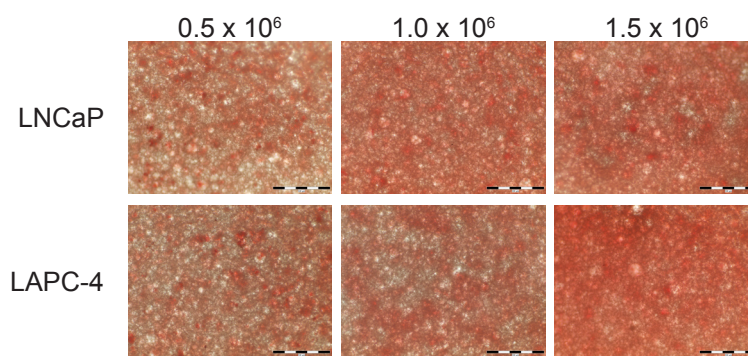

B

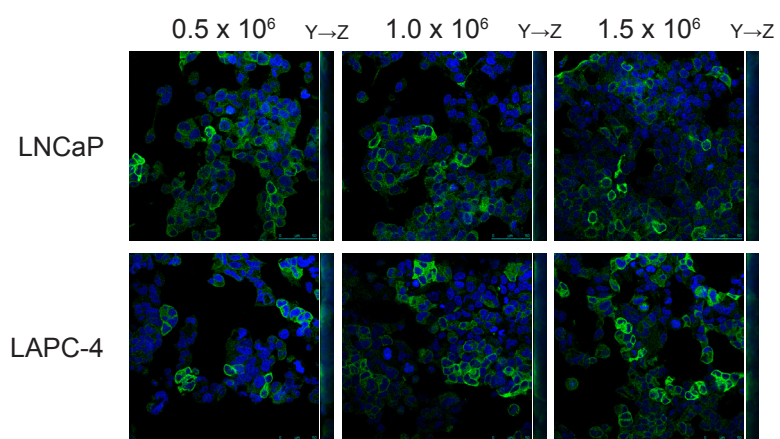

C

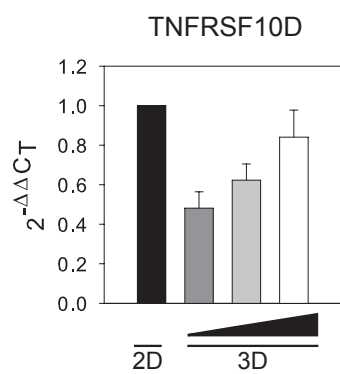

Supplement: Additional file 2: Figure S2 — Cultivation of prostate cancer cell lines in 3D conditions using Alvetex scaffold. A, LNCaP and LAPC-4 cells were cultivated in 3D conditions using Alvetex® scaffold at the indicated seeding densities per insert in complete media. After 72 hours, live cells were visualized by staining with 0.5% neutral red solution (N6634, Sigma-Aldrich) according to the manufacturer’s protocol. Increased intensity of staining indicates increased cell density. B, Immunofluorescence detection of tubulin β-III expression in LNCaP and LAPC-4 cells after 3 days of cultivation on Alvetex® inserts. Staining was performed according to the manufacturer’s protocol. Specifications of the antibodies used are provided in Table S2. C, qRT-PCR analysis of DcR2 gene (TNFRSF10D) in PC3-AR cells cultivated in 3D conditions on Alvetex scaffold as described in Additional file 9. The triangle represents increasing seeding density in 3D conditions on Alvetex (0.5×10^6^, 1.0×10^6^, and 1.5×10^6^, respectively). Results from two independent repetitions are presented (n=2). [file 1476-4598-13-113-S2.pdf]

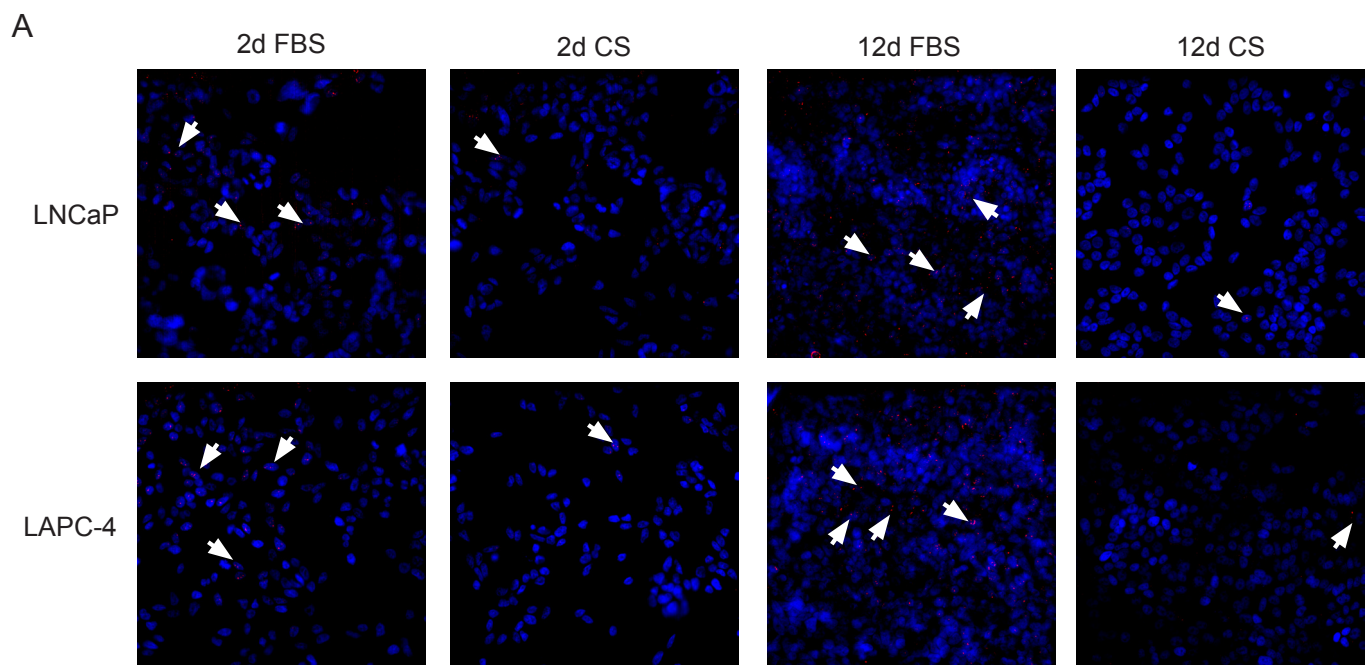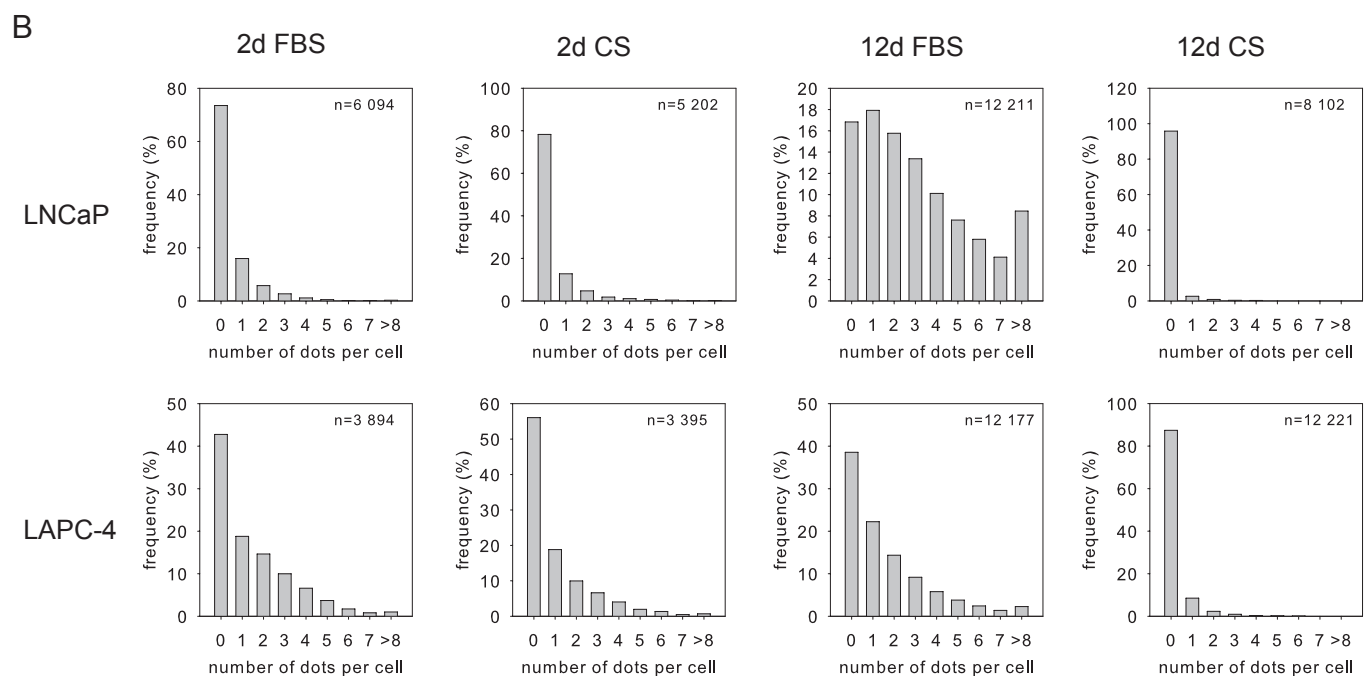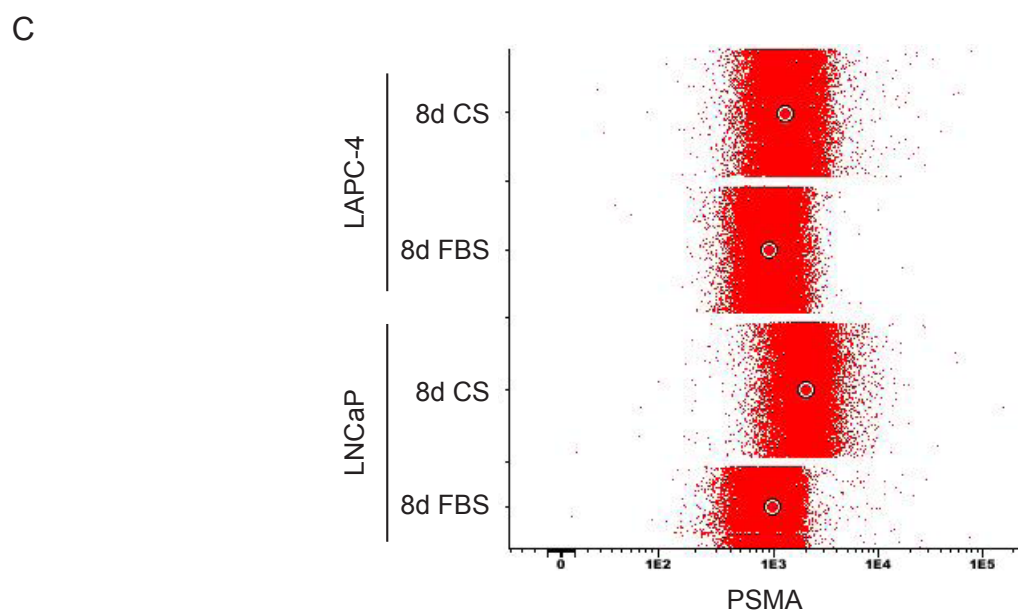

Supplementary Figure 3

Supplement: Additional file 3: Figure S3 — Assessment of AR activity at a single cell level after high-density cultivation and prolonged androgen ablation. A, Activity of AR in response to androgen depletion (12d CS) and at high density (12d FBS) assessed by detection of KLK3 mRNA using a mRNA FISH technique and quantified (B) as described in Additional file 9. n, number of identified nuclei C, Flow cytometric analysis of prostate membrane specific antigen (PSMA) in LNCaP and LAPC-4 cells in response to androgen depletion (8d CS) or high density (8d FBS). Staining was performed as described in Additional file 9. Representative results from one repetition out of two performed in replicate are presented. d, days. [file 1476-4598-13-113-S3.pdf]

**A**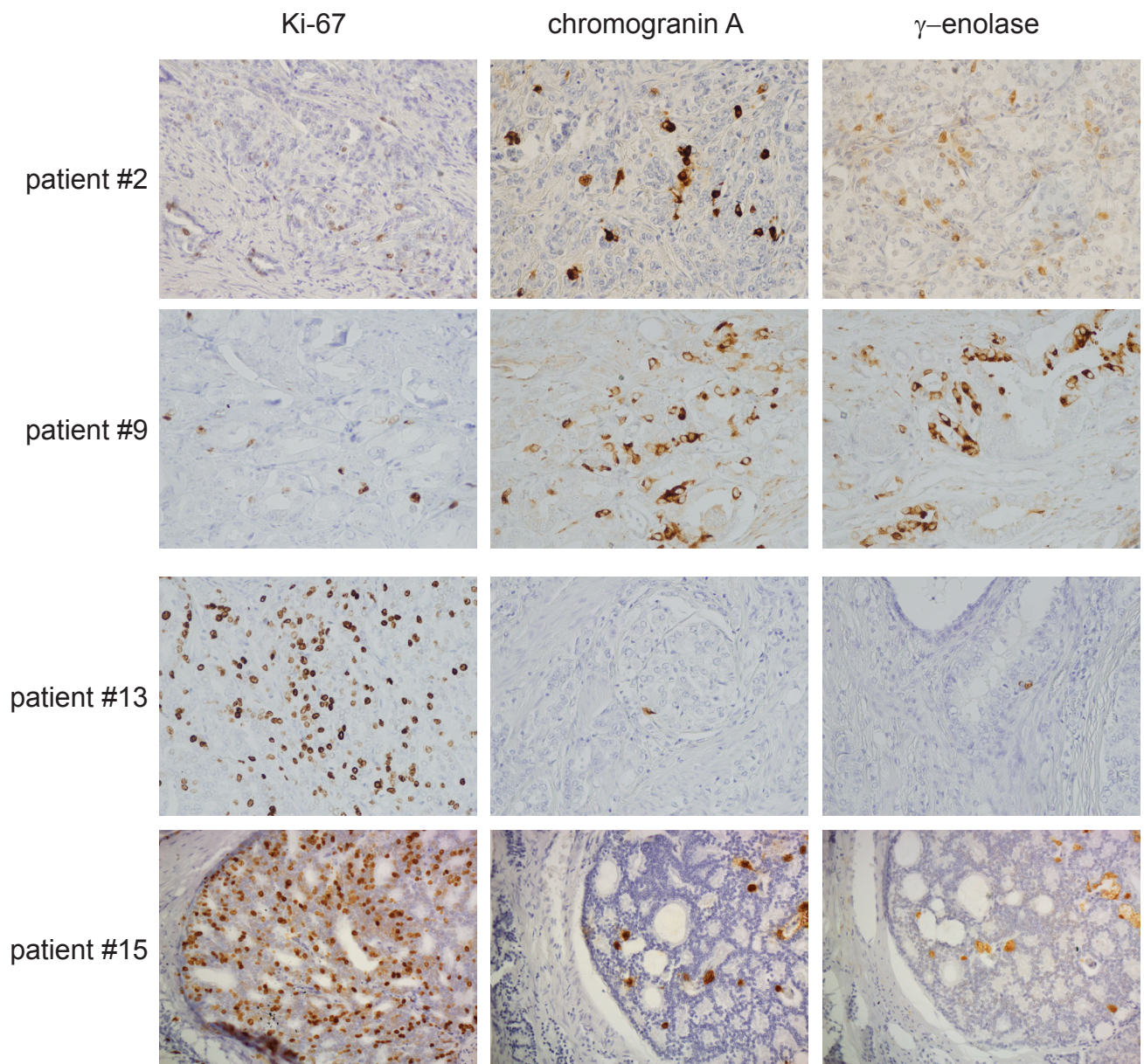**B**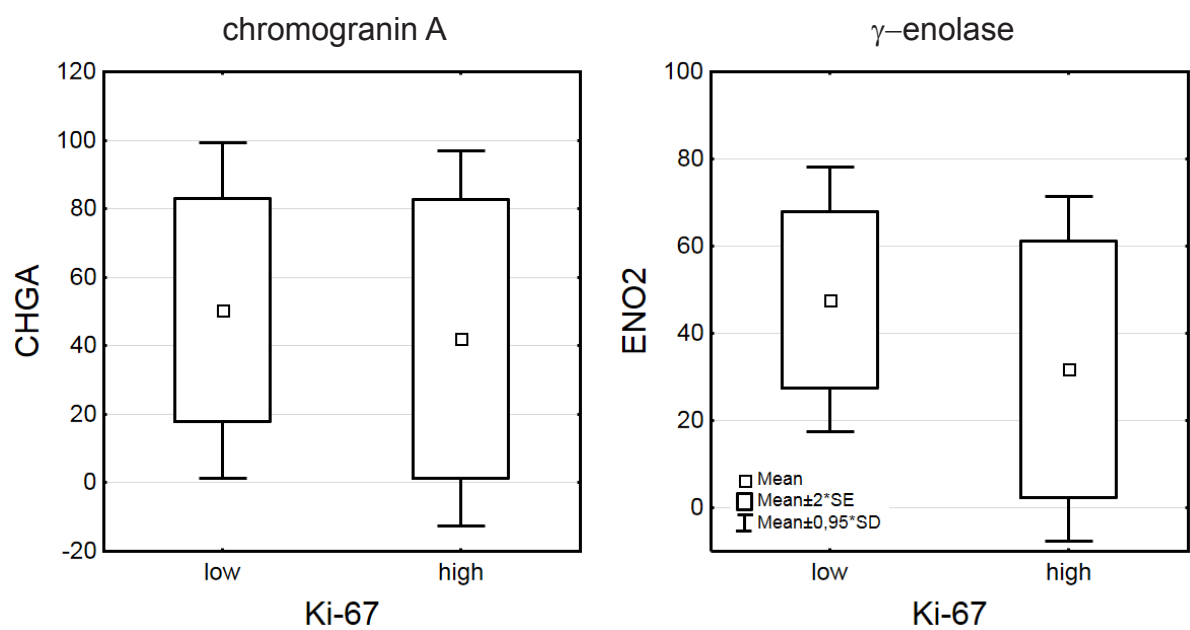

Supplement: Additional file 5: Figure S4 — Immunohistochemical staining of formalin-fixed paraffin-embedded patient samples. A, Patients 2 and 9 display low Ki-67 expression (less than 30% nuclear positivity) and multiple chromogranin A- and γ-enolase-positive NE and/or NE-like cells. Patients 13 and 15 display high Ki-67 expression (more than 30% nuclear positivity) and single chromogranin A and γ-enolase-positive NE and/or NE-like cells (magnification 40×). B, Quantification of γ-enolase and chromogranin A expression in patient tumor samples. In total, 10 patients with low Ki-67 expression and 8 patients with high Ki-67 expression were examined. Information on the patients is provided in Table S3. [file 1476-4598-13-113-S5.pdf]

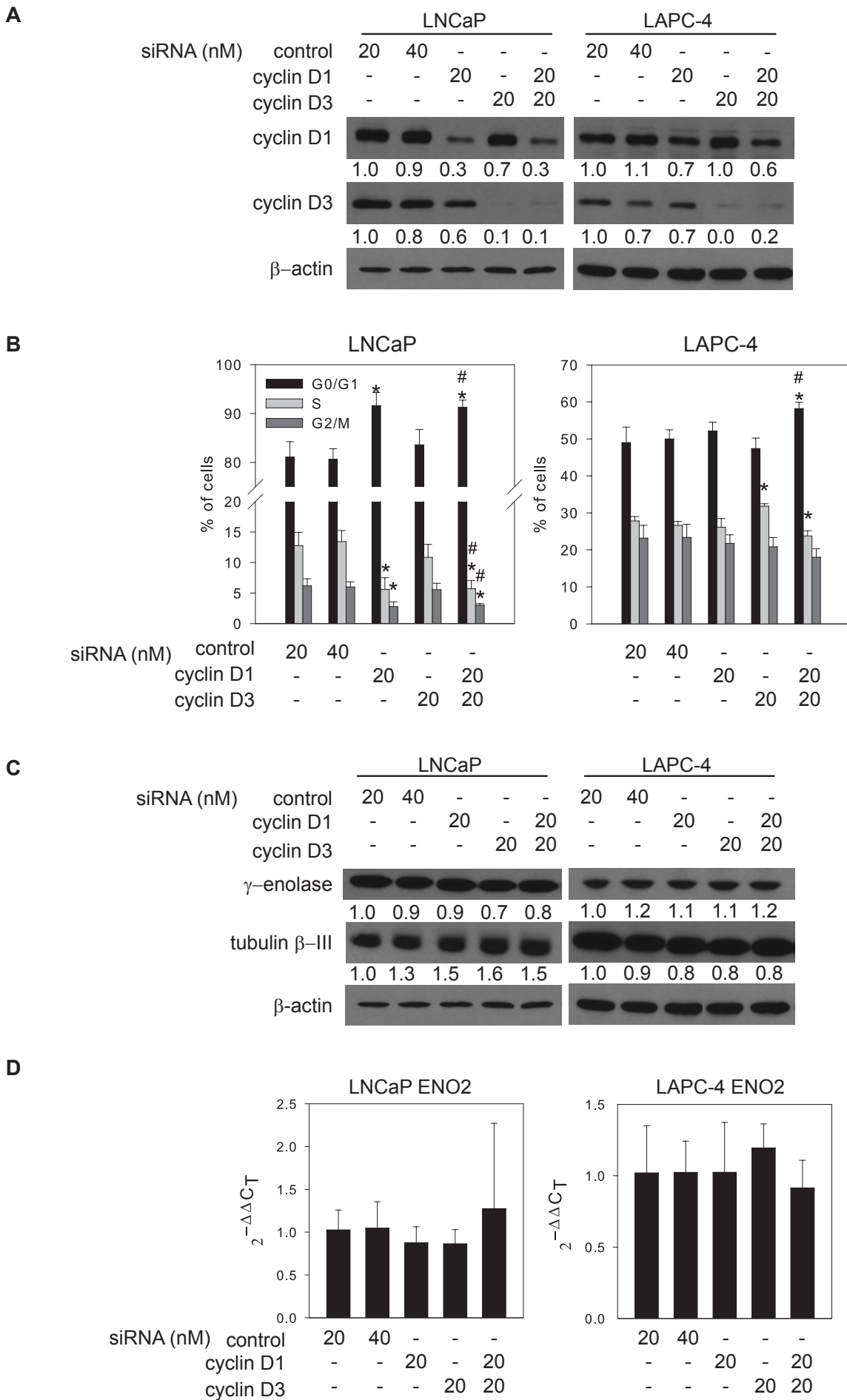

Supplementary Figure 5

Supplement: Additional file 6: Figure S5 — Modulation of the cell cycle by down-regulation of cyclin D1 and/or cyclin D3 does not lead to induction of NED in LNCaP and LAPC-4 cells. A, Western blot analysis of the efficiency of cyclin D1 and cyclin D3 down-regulation in LNCaP and LAPC-4 cells following transfection with control siRNA A or specific siRNA (20 or 40 nM). Experiments were performed as described in Supplementary Materials and Methods. B, Analysis of changes in cell cycle distribution in response to cyclin D1 and/or cyclin D3 down-regulation in LNCaP and LAPC-4 cells. Data represent means ± SD of three independent experiments. C, Western blot analysis of changes in protein levels of NED markers in response to cyclin D1 and/or cyclin D3 siRNA. It should be noted that other samples irrelevant to this study were analyzed on the same membranes. These samples were omitted from the pictures presented and the western blots presented here are therefore cropped. D, qRT-PCR analysis of changes in mRNA level of the NED marker γ-enolase (ENO2) in response to cyclin D1 and/or cyclin D3 siRNA. Data represent means ± SD of two independent experiments performed in duplicate (n=4). “*” and “#” denote statistical significance (P<0.05) compared with cells transfected with 20 nM or 40 nM control siRNA A, respectively. [file 1476-4598-13-113-S6.pdf]
