# Supplementary material for: The role of high cell density in the promotion of neuroendocrine transdifferentiation of prostate cancer cells
Source: Mol Cancer. 2014 May 20;13:113. doi: 10.1186/1476-4598-13-113 (PMC4229954; doi:10.1186/1476-4598-13-113)
Supplement: Additional file 4: Table S3 — Characteristics of human prostate tumor samples. Subsets of patients with advanced CaP with lymph node metastases were selected to obtain a sufficient percentage of NED for statistical evaluation. dg, diagnosis; GS, Gleason score; pT, pathologic T stage; pN, positivity of lymph nodes; %, percentage of positive staining. [file 1476-4598-13-113-S4.docx]

Table S3: Characteristics of human prostate tumor samples

| Patient | age | iPSA | Gleason score | pT | pN | neoadjuvant therapy | Ki67 nucl% | Ki67 | ENO2 % | ENO2 intensity | ENO2 histoscore | CHGA % | CHGA intensity | CHGA histoscore |
| --- | --- | --- | --- | --- | --- | --- | --- | --- | --- | --- | --- | --- | --- | --- |
| 1 | 64 | 15,6 | 7 | pT2c | pN1 | none | 0 | low | 40 | 2 | 80 | 5 | 3 | 15 |
| 2 | 63 | 24,0 | 7 | pT3b | pN1 | none | 10 | low | 40 | 2 | 80 | 40 | 3 | 120 |
| 3 | 64 | 13,6 | 8 | pT4 | pN1 | none | 10 | low | 30 | 1 | 30 | 50 | 3 | 150 |
| 4 | 67 | 12,3 | 9 | pT3b | pN1 | none | 10 | low | 3 | 2.5 | 7.5 | 3 | 3 | 9 |
| 5 | 61 | 25,0 | 7 | pT2c | pN1 | none | 10 | low | 10 | 2.5 | 25 | 15 | 3 | 45 |
| 6 | 56 | 28,0 | 9 | pT3b | pN1 | bicalutamide | 10 | low | 10 | 1.5 | 15 | 5 | 3 | 15 |
| 7 | 70 | 11,0 | 9 | pT3b | pN1 | none | 10 | low | 10 | 2 | 20 | 0 | 0 | 0 |
| 8 | 60 | 9,5 | 9 | pT3b | pN1 | none | 20 | low | 40 | 2 | 80 | 30 | 3 | 90 |
| 9 | 63 | 41,0 | 7 | pT3b | pN1 | none | 20 | low | 30 | 3 | 90 | 10 | 3 | 30 |
| 10 | 57 | 2,8 | 7 | pT3a | pN1 | none | 25 | low | 20 | 2.5 | 50 | 10 | 3 | 30 |
| 11 | 62 | 15,4 | 7 | pT4 | pN1 | none | 30 | high | 20 | 2 | 40 | 15 | 3 | 45 |
| 12 | 61 | 8,2 | 8 | pT4 | pN1 | none | 35 | high | 0 | 0 | 0 | 5 | 3 | 15 |
| 13 | 59 | 40,0 | 7 | pT3b | pN1 | none | 40 | high | 2 | 1 | 2 | 2 | 2 | 4 |
| 14 | 69 | 10,3 | 9 | pT3b | pN1 | none | 40 | high | 0 | 0 | 0 | 15 | 2 | 30 |
| 15 | 64 | 12,2 | 8 | pT3b | pN1 | none | 50 | high | 5 | 3 | 15 | 10 | 3 | 30 |
| 16 | 68 | 33,2 | 7 | pT3b | pN1 | none | 50 | high | 50 | 1,5 | 75 | 10 | 3 | 30 |
| 17 | 76 | 30,7 | 9 | pT3b | pN1 | none | 60 | high | 4 | 2.5 | 10 | 1 | 3 | 3 |
| 18 | 63 | 9,4 | 9 | pT3b | pN1 | none | 70 | high | 45 | 2.5 | 112.5 | 60 | 3 | 180 |

age, age at diagnosis; iPSA, initial serum PSA; pT, pathologic T stage; pN, positivity of lymph nodes; %, percentage of staining positivity
