# Supplementary material for: The role of high cell density in the promotion of neuroendocrine transdifferentiation of prostate cancer cells
Source: Mol Cancer. 2014 May 20;13:113. doi: 10.1186/1476-4598-13-113 (PMC4229954; doi:10.1186/1476-4598-13-113)
Supplement: Additional file 7: Table S1 — Sequences of primers used in quantitative RT-PCR. [file 1476-4598-13-113-S7.docx]

| **A) sequences of primers used in one-step qRT-PCR with TaKaRa One Step Syber Kit** | | |
| --- | --- | --- |
| **Gene** | **Oligonucleotide** | **Accession No.** |
| **DDC**  Homo sapiens aromatic L-amino-acid decarboxylase | F: TGTCCAGCTGTCCCATGAGTT R: CAGAATGACTTCCACACAGATTTCA | NM_000790  NM_[001082971](http://www.ncbi.nlm.nih.gov/sites/entrez?db=nuccore&cmd=search&term=NM_001082971) |
| **CHGA**  Homo sapiens chromogranin-A | F: GCGGTGGAAGAGCCATCAT R: TCTGTGGCTTCACCACTTTTCTC | NM_001275 |
| **ENO2**  Homo sapiens gamma-enolase | F: CCCTGTATCGCCACATTGC R: GCCACCATTGATCACGTTGA | NM_001975 |
| **KLK3**  Homo sapiens prostate-specific antigen | F: CCTCCTGAAGAATCGATTCCT R: CGTCCAGCACACAGCATGAA | [NM_001030047](http://www.ncbi.nlm.nih.gov/sites/entrez?db=nuccore&cmd=search&term=NM_001030047) [NM_001030048](http://www.ncbi.nlm.nih.gov/sites/entrez?db=nuccore&cmd=search&term=NM_001030048)  [NM_001030049](http://www.ncbi.nlm.nih.gov/sites/entrez?db=nuccore&cmd=search&term=NM_001030049) [NM_001030050](http://www.ncbi.nlm.nih.gov/sites/entrez?db=nuccore&cmd=search&term=NM_001030050)  [NM_001648](http://www.ncbi.nlm.nih.gov/sites/entrez?db=nuccore&cmd=search&term=NM_001648) |
| **POLR2A**  Homo sapiens polymerase (RNA) II (DNA directed) polypeptide A | F: ATCTCTCCTGCCATGACACC R: AGACCAGGCAGGGGAGTAAC | NM_000937 |
| **TUBB3**  Homo sapiens tubulin, beta 3 class III | F: AGCAAGAACAGCAGCTACTTCGT R: GATGAAGGTGGAGGACATCTTGA | NM_001197181.1 |

**Table S1: Sequences of primers used in quantitative RT-PCR**

| **B) sequences of primers used in two-step qRT-PCR with UPL system** | | | | |
| --- | --- | --- | --- | --- |
| **Gene** | **Oligonucleotide** | **UPL probe** | **Assay Efficiency** | **Accession No.** |
| **ENO2**  Homo sapiens gamma-enolase | F: ctgtggtggagcaagagaaa  R: acacccaggatggcattg | #67 | 2.022 | NM_001975.2 |
| **KLK3**  Homo sapiens kallikrein-related peptidase 3 (KLK3), transcript variant 1 | F: gtgcttgtggcctctcgt R: agcaagatcacgcttttgttc | #44 | 1.965 | NM_001648.2 |
| **POLR2A**  Homo sapiens polymerase (RNA) II (DNA directed) polypeptide A | F: GCAAATTCACCAAGAGAGACG  R: CACGTCGACAGGAACATCAG | #1 | 2.062 | NM_000937.3 |
| **TNFRSF10D**  Homo sapiens tumor necrosis factor receptor superfamily, member 10d, decoy with truncated death domain | F: gcctcaaggaggaggagtg R: tctgtgcacgggttacagg | #18 | 1.860 | NM_003840.3 |
| **TUBB3**  Homo sapiens tubulin, beta 3 class III | L: gcaactacgtgggcgact R: atggctcgaggcacgtact | #78 | 2.417 | ENST00000554444.1\|  ENSG00000198211.7 |
