# Supplementary material for: The role of high cell density in the promotion of neuroendocrine transdifferentiation of prostate cancer cells
Source: Mol Cancer. 2014 May 20;13:113. doi: 10.1186/1476-4598-13-113 (PMC4229954; doi:10.1186/1476-4598-13-113)
Supplement: Additional file 8: Table S2 — Specification of antibodies used for western blot analysis, immunofluorescence and flow cytometry. [file 1476-4598-13-113-S8.docx]

| **Table S2:**  **Antibodies for western blotting** | | | |
| --- | --- | --- | --- |
| **Protein** | **Vendor** | **Catalog No.** | **Dilution** |
| AR | Santa Cruz | sc-815 | 1:1 000 |
| Cdk1 (cdc2 p34) | Santa Cruz | sc-954 | 1:1 000 |
| Cdk2 | Santa Cruz | sc-163 | 1:500 |
| p-Cdk2 (Thr160) | Cell Signaling Technology | 2561 | 1:500 |
| CREB | Cell Signaling Technology | 9197 | 1:500 |
| p-CREB (Ser133) | Cell Signaling Technology | 9198 | 1:500 |
| cyclin D1 | Santa Cruz | sc-20044 | 1:500 |
| cyclin D3 | Santa Cruz | sc-182 | 1:500 |
| DcR2 | BD Pharmingen | 68861N | 1:1 000 |
| DcR2 | Sigma | D3188 | 1:1 000 |
| γ-enolase | Santa Cruz | sc-21738 | 1:500 |
| PKA RII (Ser96) | Epitomics | 1151 | 1:500 |
| p-(Ser/Thr) PKA substrate | Cell Signaling Technology | 9612 | 1:500 |
| Rb | BD Biosciences | 554136 | 1:500 |
| p-Rb (Ser807/811) | Santa Cruz | sc-16670 | 1:500 |
| p-Rb (Ser807/811) | Cell Signaling | 9308S | 1:1 000 |
| tubulin β-III | Promega Corporation | G7121 | 1:500 |
| p27^Kip1^ | BD TL | 610242 | 1:1 000 |
| β-actin | Sigma-Aldrich | A5441 | 1:4 000 |
| α-tubulin | Sigma-Aldrich | T9026 | 1:4 000 |
| anti-mouse IgG | GE Healthcare Biosciences | NA931 | 1:4 000 |
| anti-rabbit IgG | GE Healthcare Biosciences | NA934 | 1:3-6 000 |
| **Antibodies for immunofluorescence microscopy** | | | |
| tubulin β-III | Promega Corporation | G7121 | 1:200 |
| donkey anti-mouse  Alexa Fluor 488 | Life Technologies | A-21202 | 1:2 000 |
| 4',6-Diamidino-2-phenylindole dihydrochloride (DAPI 1mg/ml) | AppliChem | A4099,0025 | 1:1 000 |
| TO-PRO-3 iodid 1mM | Life Technologies | T 3605 | 1:1 000 |
| **Antibodies for immunohistochemistry** | | | |
| anti-NSE | Serotec | BBS/NC/V1-H14 | Ready-to-use |
| chromogranin A | Serotec | LK2H10 | Ready-to-use |
| Ki-67 | Dako | MIB-1 | 1:25 |
| secondary antibody | Dako | Dual Link | Ready-to-use |
| **Antibodies for flow cytometry** | | | |
| PSMA | Abcam | ab19071 | 1:200 |
| donkey anti-mouse  Alexa Fluor 488 | Life Technologies | A-21202 | 1:2 000 |
