# Supplementary material for: The role of high cell density in the promotion of neuroendocrine transdifferentiation of prostate cancer cells
Source: Mol Cancer. 2014 May 20;13:113. doi: 10.1186/1476-4598-13-113 (PMC4229954; doi:10.1186/1476-4598-13-113)
Supplement: Additional file 9 — Supplementary Material and Methods. [file 1476-4598-13-113-S9.docx]

**Supplementary Material and Methods**

**Promotion of NED by high density in PC3, DU-145, and PC3-AR cells**

PC3, PC3-AR, and DU-145 cells were cultivated as described in Material and Methods. For high density–induced NED, cells were seeded at standard density (20,000/cm^2^ of cultivation surface) and cultivated for 2 days or 10 days. During this period the cells were not split, but the medium was exchanged for appropriate complete growth medium twice a week. Experiments were performed twice in duplicate (n=4).

**mRNA FISH**

LNCaP and LAPC-4 cells were seeded on μ-Slide 8 wells (Ibidi) and cultured for NED induction as described in Material and Methods for 2 (2d) and 12 (12d) days. After cultivation, cells were fixed in 3.7% paraformaldehyde in PBS and stored in 70% EtOH at 4°C for up to 1 week. The mRNA level of the KLK3 gene was assessed using the Stellaris® FISH Probe KLK3 with Quasar® 670 Dye (SMF-1065-5, sequence:

cacatggtgacacagctctcacagggtgaggaagacaacccaatccgagacaggatgagggcaagatcacgcttttgttccctgtgtcttcaggatgaaactgtggctgacctgaaatacattcttcaggaggctcatattccatgaccttcacagcatcagtttctttggggtcaagaaaacatggaggtccacacactgcgcacacacgtcattggaagtcaccttctgagggtgaactccagcacacagcatgaactgtcagttcggtgatcagaatcaggactactctctgactagaggacagtccttgtagacaggacttccagaagaaggtgggctggacaggcagataacagatgggcaatcctatctttcaggggagagagtgagataggtccagaatcaccctaagggtaacaccattacagacaagtgggccatgacgtgataccttgaacaccttggtgtacagggaagttgatccacttccggtaatgacaatagggggttgatagggatttccaaggttccaagtttacctcacacctaaggacaaagaaacactctggtctacaccctatctgtgtcctcagagaaatagtgaatcagcttccagg). Samples were processed according to the manufacturer’s instructions; the hybridization probe for KLK3 was used at a final concentration of 0.25 μM. After hybridization and washing, slides were mounted in anti-fade Glox buffer with catalase and glucose oxidase. Analysis was performed immediately after mounting using Image Xpress MicroXL (Molecular Devices) with a 40× S Plan Fluor EL, WD> 2 mm, 0.60 NA objective. The following filters were used: MolDev 1-6300-0442 FILSTER SET, ZPS, DAPI (excitation/emission 377/447) and MolDev 1-6300-0446 FILTER SET, ZPS, CY5 (excitation/emission 628/692). Quantitative image analysis was accomplished for KLK3 expression. The ratios of RNA foci number per nuclei were count using CellProfiler Software (http://www.cellprofiler.org/). The images were acquired with n = 2 biological replicate wells per condition, each well analyzed in an average of ten images (~500 nuclei per image). The algorithm was based on selection of cell nuclei used as the mask for RNA channel. Primary objects/nuclei were identified by DAPI staining, and the number of RNA foci inside the primary object was measured. The Gaussian and FFT bandpass filter were applied to suppress noise of the RNA images using ImageJ (http://imagej.nih.gov/ij/). The threshold for RNA foci detection of cells cultured in FBS and CS medium was set the same for each cell line and cultivation period. The results were evaluated as frequency of foci count per nuclei.

**Flow cytometric analysis of prostate specific membrane antigen (PSMA)**

LNCaP and LAPC-4 cells were cultured in medium with FBS or CS for 8 days (8d) as described in Material and Methods. Cells were trypsinized, fixed in 2% paraformaldehyde, permeabilized with 0.1% TritonX-100 for 15 min, and incubated with anti-PSMA primary antibody followed by incubation with anti-mouse IgG Alexa 488 secondary antibody. PSMA expression was measured using FACSVerse (Becton Dickinson). Data were analyzed using Infinicyt^TM^ flow cytometry software (Cytognos). Specifications of the antibodies used are provided in Table S2.

**Immunostaining of human prostate cancer cells**

Formalin-fixed, paraffin-embedded (FFPE) human prostate tumor samples were obtained from 18 patients with lymph node metastases. Samples were immunostained with appropriate antibodies (listed in Table S2) using routine methods. Ki-67 was assessed by the percentage of nuclear positivity whereas ENO2 and CHGA were assessed by histoscore (percentage of positivity in the area of interest multiplied by staining intensity categorized as follows: 0, absent; 1 weak; 2, moderate; and 3, strong). Patients with lymph node metastasis were selected from a larger study (unpublished) that was approved by the ethics committee of the Faculty of Medicine and Dentistry, Palacky University, Olomouc.

**RNA interference**

LNCaP and LAPC-4 cells were transfected with small inhibitory RNA (siRNA) duplexes (Santa Cruz Biotechnology) directed against cyclin D1 (sc-44257), cyclin D3 (sc-35136), or non-targeting control (sc-37007) as described previously [38]. Three independent experiments (cell cycle) or two experiments in duplicate (mRNA, n=4) were performed.

**Supplementary figure legends**

**Supplementary Figure 1**

**A, Induction of neuroendocrine transdifferentiation by high cell density, but not by androgen depletion, is a reversible process**. Cells were cultivated for 16 days to induce NED as described in Material and Methods section. After 16 days the cells were re-seeded at a low density (10,000 cells/cm^2^) in the appropriate cultivation media. Cells grown in FBS were re-seeded into media with FBS; cells grown in CS were re-seeded into either CS or FBS. Cells were further cultivated for 2, 4, and 8 days (16+2, 16+4, and 16+8, respectively) without splitting and appropriate medium was exchanged with fresh one twice a week. Expression of the NED marker γ-enolase in response to re-seeding was assessed using western blot analysis. A typical result of three independent repetitions is presented. **B-D, NED is promoted by high density also in AR-negative prostate epithelial cell lines.** B, PC-3, PC3-AR, and DU-145 cells were cultivated as described in Supplementary Material and Methods. Expression of NED markers γ-enolase and tubulin β-III was assessed by western blot analysis. C, Western blot analysis of AR expression to confirm its presence in PC3-AR cells; LNCaP cells served as a positive control. Results from one repetition out of two performed in technical duplicate are presented. D, qRT-PCR analysis of the NED marker γ-enolase (ENO2) and tubulin β-III (TUBB3) in PC3, PC3-AR, and DU-145 cells cultivated as described. Results from two repetitions performed in technical duplicate are presented (n=4). **E,** qRT-PCR analysis of DcR2 gene (TNFRSF10D) in PC3-AR cells cultivated as described in Supplementary Material and MethodsResults from two repetitions performed in technical duplicate are presented (n=4).

**Supplementary Figure 2**

**Cultivation of prostate cancer cell lines in 3D conditions using Alvetex scaffold.**

A, LNCaP and LAPC-4 cells were cultivated in 3D conditions using Alvetex® scaffold at the indicated seeding densities per insert in complete media. After 72 hours, live cells were visualized by staining with 0.5% neutral red solution (N6634, Sigma-Aldrich) according to the manufacturer´s protocol. Increased intensity of staining indicates increased cell density. B, Immunofluorescence detection of tubulin β-III expression in LNCaP and LAPC-4 cells after 3 days of cultivation on Alvetex® inserts. Staining was performed according to the manufacturer´s protocol. Specifications of the antibodies used are provided in Table S2. C, qRT-PCR analysis of DcR2 gene (TNFRSF10D) in PC3-AR cells cultivated in 3D conditions on Alvetex scaffold as described in Supplementary Material and Methods. The triangle represents increasing seeding density in 3D conditions on Alvetex (0.5×10^6^, 1.0×10^6^, and 1.5×10^6^, respectively). Results from two independent repetitions are presented (n=2).

**Supplementary Figure 3**

**Assessment of AR activity at a single cell level after high-density cultivation and prolonged androgen ablation.**

A, Activity of AR in response to androgen depletion (12d CS) and at high density (12d FBS) assessed by detection of KLK3 mRNA using a mRNA FISH technique and quantified (B) as described in Supplementary Material and Methods. n, number of identified nuclei C, Flow cytometric analysis of prostate membrane specific antigen (PSMA) in LNCaP and LAPC-4 cells in response to androgen depletion (8d CS) or high density (8d FBS). Staining was performed as described in Supplementary Material and Methods. Representative results from one repetition out of two performed in replicate are presented. d, days.

**Supplementary Figure 4**

**Immunohistochemical staining of formalin-fixed paraffin-embedded patient samples.**

A, Patients 2 and 9 display low Ki-67 expression (less than 30% nuclear positivity) and multiple chromogranin A- and γ-enolase-positive NE and/or NE-like cells. Patients 13 and 15 display high Ki-67 expression (more than 30% nuclear positivity) and single chromogranin A and γ-enolase-positive NE and/or NE-like cells (magnification 40×). B, Quantification of γ-enolase and chromogranin A expression in patient tumor samples. In total, 10 patients with low Ki-67 expression and 8 patients with high Ki-67 expression were examined. Information on the patients is provided in Table S3.

**Supplementary Figure 5**

**Modulation of the cell cycle by down-regulation of cyclin D1 and/or cyclin D3 does not lead to induction of NED in LNCaP and LAPC-4 cells.**

A, Western blot analysis of the efficiency of cyclin D1 and cyclin D3 down-regulation in LNCaP and LAPC-4 cells following transfection with control siRNA A or specific siRNA (20 or 40 nM). Experiments were performed as described in Supplementary Materials and Methods. B, Analysis of changes in cell cycle distribution in response to cyclin D1 and/or cyclin D3 down-regulation in LNCaP and LAPC-4 cells. Data represent means ± SD of three independent experiments. C, Western blot analysis of changes in protein levels of NED markers in response to cyclin D1 and/or cyclin D3 siRNA. It should be noted that other samples irrelevant to this study were analyzed on the same membranes. These samples were omitted from the pictures presented and the western blots presented here are therefore cropped. D, qRT-PCR analysis of changes in mRNA level of the NED marker γ-enolase (ENO2) in response to cyclin D1 and/or cyclin D3 siRNA. Data represent means ± SD of two independent experiments performed in duplicate (n=4). “*” and “^#^” denote statistical significance (P<0.05) compared with cells transfected with 20 nM or 40 nM control siRNA A, respectively.

**Table S1: Sequences of primers used in quantitative RT-PCR**

**Table S2: Specification of antibodies used for western blot analysis, immunofluorescence and flow cytometry**

**Table S3: Characteristics of human prostate tumor samples**

Subsets of patients with advanced CaP with lymph node metastases were selected to obtain a sufficient percentage of NED for statistical evaluation. dg, diagnosis; GS, Gleason score; pT, pathologic T stage; pN, positivity of lymph nodes; %, percentage of positive staining.
